# Drying characteristics and quality enhancement of *Moringa oleifera* seeds through Ultrasound and Microwave-Assisted germination combined with infrared vacuum drying

**DOI:** 10.1016/j.ultsonch.2025.107432

**Published:** 2025-06-15

**Authors:** Palwasha Gul, Jabir Khan, Yang Li, Qingyun Li, Huiyan Zhang, Kunlun Liu

**Affiliations:** a College of Food Science and Engineering, Henan University of Technology, Zhengzhou 450001, China; b Mengniu Institute of Nutrition Science, Global R&D Innovation Center, Inner Mongolia Mengniu Dairy (Group) Co., Ltd., Beijing 101107, China; c Zhengzhou Ruipu Biological Engineering Co., Ltd., Zhengzhou 450001, China

**Keywords:** *Moringa oleifera*, Pre-treatments assisted germination, Infrared vacuum drying, Mathematical modelling, Nutritional profile, Antioxidant capacity, GC–MS analysis

## Abstract

*Moringa oleifera* (MO), is greatly appreciated for its high nutritional profile. Germination is an efficient technique to enhance the quality profile of seeds. This study examined the effects of germination and various pre-treatments on MO seeds. MO seeds were subjected to ultrasound dual-frequency 10 and 20 min (US 10 & US 20), microwave 30 and 60 s (MW 30 & MW 60), followed by 15-day germination at 25 °C in a biochemical incubator and subsequent infrared vacuum drying at 70, 60 and 50 °C. Several mathematical modelling were applied, among which Midilli-Kucuk model showed excellent fit R^2^ ≥ 0.9997, X^2^ ≤ 0.0030, RMSE ≤ 0.0010, and RSS ≤ 0.000, followed by Newton model respectively. The efficiency of the process varied across different treatment conditions, with values ranging from 3.4 % to 10.27 %, indicating significant differences in energy consumption and water removal rates. Overall in the subsequent drying, US 10 at 60 °C significantly improved quality profile of MO seeds (p ≤ 0.05). Antioxidant potency composite (APCI) of germinated MO seeds showed superior APCI in US 10 dried at 60 °C (97.50 %) than control (56.55 %). The principal component analysis (PCA) revealed a significant correlation matrix, explaining 62.14 % of the total variability in the nutritional and bioactive components of MO seed. GC–MS analysis revealed 50 volatile compounds, categorized into 9 chemical classes with acids being the most abundant. SEM analysis showed that 70 °C, drying caused structural degradation, whereas 60 °C preserved seed microstructure more effectively, revealed pre-treatments and temperature-dependent microstructural alterations. These findings demonstrate the efficacy of pre-treatments assisted germination and moderate-temperature infrared vacuum drying as a viable option for improving the nutritional profile of MO seeds. Importantly, this study did not employ formal optimization techniques such as response surface methodology (RSM), energy cost minimization, full techno-economic and life cycle assessments from an engineering perspective. Instead, it sufficiently explored the drying kinetics and the impact of different pre-treatments on the quality profile of MO seeds.

## Graphical abstract

**Plan of the study**, Ultrasound dual frequency 40+40 kHz for 10 and 20 min (Ultrasound 10, 20 mins); Biochemical Incubator (B.I); Water consumption (W.C); Energy consumption (E.C); Drying rate (D.R); Efficiency (ή); Drying chamber (1); Centrifugal fan (2); Infrared quartz glass tubes (3); Wire-mesh tray (4); Handling unit (5); Air controller (6); Door (7); Monitoring display (8); Temperature controller (9); Vacuum controller (10); Dryer controller (11).

**Figure:**
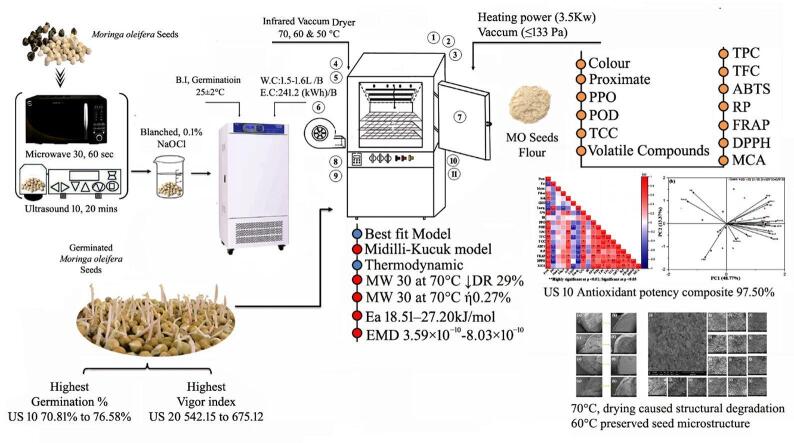

## 1 Introduction

*Moringa Oleifera* (MO), a fast-growing tree, drought-resistant plant, often called “Moringa,” “drumstick,” “sahjan,”, “horseradish” referred, as a superfood are beneficial for human health [1]. MO seeds are rich in nutritional values & are used in traditional medicine to cure anaemia, diarrhoea, constipation, & several chronic non-communicable diseases [2]. MO seeds contain 28.01–32.21 % protein, fat 28.73–31.9 % [3,4], fibre 3.11–5.20 %, and carbohydrate 10.56–22.67 % [3,5]. Germination process may regarded as a good strategy for enhancing the nutritional value of grain seeds [6]. Numerous studies have demonstrated that controlled germination, as a biotechnological process, significantly improves the nutritional profile of seeds [7,8]. The biochemistry and physiology of seeds are notably influenced by factors such as pre-treatment methods, temperature, and time. Consequently, various pre-treatment techniques like Ultrasound (US) and Microwave (MW), commonly referred to as seed enhancers, have been used to improve seed germination rates and other quality attributes [9,10]. To significantly improve the quality of MO seeds, pre-treatments before germination are recommended. However, the effects of these treatments on seed quality can vary depending on the specific method employed [11]. Recent research has explored the application of these technologies for enhancing seed quality in various crops, including MO leaves [12], sorghum [13], soybean [14], and quinoa [10]. Despite these advancements, no study has comprehensively examined the combined effects of dual-frequency US and MW pre-treatments with varying durations on controlled germination, followed by drying at different temperatures. Additionally, the influence of these treatments on the detailed quality characteristics of MO seeds remains unexplored. Therefore, it is crucial to conduct an in-depth study to evaluate whether these pre-treatment techniques alter the quality profile of MO seeds.

In modern era, researchers have focused on alternatives to enhance product durability and minimize quality loss during long-term storage [4]. Drying, an ancient preservation method, reduces moisture content, inhibits enzymatic and microbial activity, and lowers product weight and volume [5]. Various drying techniques, including sun-drying, hot-air, and infrared vacuum dryer, vacuum freezing, and freeze-drying, are employed, though they suffer from long processing times and high energy consumption [8]. Pre-treatments such as US and MW have been shown to reduce processing time, enhance product quality, and optimize energy efficiency [15]. Abbaspour et al. [16], demonstrated that US and MW pre-treatments reduced drying time and specific energy consumption (SEC) while improving product efficacy. Similarly, Nowacka et al. [17] reported that US pre-treatment in a microwave vacuum dryer shortened drying times for cranberry snacks while preserving bioactive components.

An examination of the scientific literature reveals that most studies on US and MW pre-treatments have focused on single-frequency ultrasound applications and microwave single power settings. Regarding the quality profile of MO, the majority of research has been conducted on MO leaves and pods, while there remains a significant gap in the exploration of MO seeds. Notably, no studies have yet examined the combined effects of dual-frequency US at 40 kHz + 40 kHz, and MW at 30 and 60 power settings on the subsequent germination and drying processes of MO seeds. The present study aims to fill this gap by investigating the effects of dual-frequency US and MW pre-treatments on drying kinetics (e.g., moisture diffusion coefficient, specific energy consumption, energy efficiency and efficnecy), germination rate, vigor index, nutritional composition, color, bioactive compound content, enzymatic activity, volatile compound profile, and structural properties of MO seeds. These effects are examined using an infrared vacuum dryer at three different temperature settings: 50 °C, 60 °C, and 70 °C. This comprehensive evaluation of MO seed quality under varying pre-treatment and drying conditions aims to provide a deeper understanding of how these treatments influence seed characteristics and structural properties.

## 2 Material and methods

### 2.1 Germination

For each experimental condition, 200 ± 2 g of MO seeds were measured in triplicate and cleaned with distilled water twice after purchase from (Henan Branch China). As per the plan after draining the water, the seeds were separated into five groups: Only germinated (OG), Microwave (30 and 60 s) and US dual frequency 40 + 40 kHz (10 and 20 min) (**Plan of the study**). For MW 30 & 60, seeds were placed in a microwave oven (G80F20CN2L-B8-R0) for 30 s and 60 s, respectively, at 300 W power. In the US group, the seeds were steeped in DI water (1:4w/v) at 25 °C, 100 % amplitude (Model: KMD-M3, Sn: 20220322027) for 10 and 20 min. Following pre-treatment, the seeds underwent blanching in a 0.1 % sodium hypochlorite solution for 20 min, followed by 2–3 rinses with deionized water. Samples from each group were then germinated at 25 ± 2°C in a biochemical incubator (Model: SPX-250B-Z11) for 15 days in replicates. Prior to the start of the experiments, an infrared vacuum dryer maintaining 3 different temperatures at once (Model: ZKT-210EF), Vacuum pressure (≤133 Pa constant across all runs), Heating power (3.5Kw),was left on for 1 h at temperatures of 70 °C, 60 °C, and 50 °C to ensure consistent drying conditions (**Process flow diagram**). After that, for each experiment conducted in the infrared chambers, the tray load density was 100 ± 2 g. In order to determine their mass on an analytical scale, samples were weighed hourly until they reached the target moisture content and remained constant in weight. Instantly after each weighing, the samples were put back into the dryer. Subsequently, the germinated and control samples were ground to a 200-mesh sieve and stored at 4 °C for further examination. To better understand the drying behaviors, various thin-layer models were employed to examine the drying kinetics of each treatment.

.

**Process flow diagram:** Ultrasound 40 + 40 kHz 10 and 20 min (US 10 & US 20); Microwave for 30 and 60 s (MW 30& MW 60); Distilled water (DI); Water consumption (W.C); Energy Consumption (E.C) Relative humidity (R.H); Moisture content (M.C); Specific Energy consumption per unit mas (SEC)**;** Water Removed (kWh/kg H_2_O removed) (WR); Drying rate g/h (DR); Efficiency % (ή).

### 2.2 Germination percentage and vigor index

Germination percentage (G%) & Vigor index (VI) was evaluated using the outline methods of El-Absy et al. [18], by using the following formula: G%= N/o of seeds germinated/total number of seeds × 100. While VI = G% x root length of germinated seedlings.

### 2.3 Mathematical Modelling

#### 2.3.1 Moisture ratio & drying rate (MR & DR)

Several mathematical models were applied on dried seeds to investigate the drying kinetics. The dry matter moisture ratio was calculated empirically for each sample. Data of moisture content from samples collected at various times and dried at various temperatures were used to calculate the MR using (**Eq.** (1) & DR (**Eq.2**)

(1) $$\mathrm{MR}=\frac{\mathrm{Mt}-Me}{\mathrm{Mo}-Me}$$

MR = moisture ratio, Mt, Mo, Me = initial, equilibrium, and initial moisture content.

(2) $$\mathrm{DR}=\frac{Mt1-Mt2}{t1-t2}$$

*Mt*1 and *Mt*2 represent the initial and final moisture contents, while t1 and t2 denote the corresponding drying times.

#### 2.3.2 Energy activation

The activation energy (Ea, KJ/mol) was estimated through Arrhenius Eq. (6) [19].

(6) $$\ln\left(k\right)=\ln\left(C\right)-\frac{\mathrm{Ea}}{\mathrm{RT}}$$

Where *C*, *Ea*, T, and R represents the Arrhenius constant, energy activation (kJ/mol), temperature (k), and general gas-constant (8.314 J/(mol K)), respectively.

#### 2.3.3 Effective moisture diffusivity (EMD)

Moisture penetration is one of the most important factors controlling the drying process. When different mechanisms are effective in transmission processes, it is difficult to examine each mechanism and measure the mass transfer rate in each one. In such processes, the description of effective diffusion is described by the Fick’s second law as follows [1,20]. One of the solutions of this partial differential equation for the one-dimensional moisture transfer in spherical geometry was proposed by Crank [21] in the form of infinite series. The EMD can be calculated using the following equation (Eq.7):

(7) $$\mathrm{MR}=\frac{\mathrm{Mt}-Me}{\mathrm{Mo}-Me}=\frac{8}{\pi^{2}}\sum_{n=1}^{\infty }{1/n}^{2}exp\left(\frac{-n^{2}\pi^{2}Defft}{r^{2}}\right)$$

Where M*t*​ is the moisture content at any time, M*o*​ is the initial moisture content, and M*e*​ is the equilibrium moisture content of the sample. The drying time is denoted by t, and D*eff*​ is the effective moisture diffusivity, r is radius of the seed. As time increases, the terms other than the first approach zero. Neglecting higher terms of the equation.

(8) $$MR=\frac{\mathrm{Mt}-Me}{\mathrm{Mo}-Me}=\frac{8}{\pi^{2}}exp\left(\frac{-\pi^{2}Defft}{r^{2}}\right)$$

Where D*eff* is obtained from the slope (K) of the graph between lnMR and drying time t. LnMR versus time resulted in a straight line with a negative slope. K is associated with D*eff* using Eq. (9)

(9) $$K=\frac{\pi^{2}Defft}{r^{2}}$$

#### 2.3.4 Total and specific energy consumption

Total energy consumption (Ec) in kWh of the Infrared vacuum dryer was calculated using the following equation (E.q 10) [1]:

(10) EnergyconsumptionEc=P×t

Where Ec represents the energy consumption in kWh, P is the power rating of the Infrared vacum dryer in kWh, and t denotes the drying time in hours.

Specific amount of specific energy consumption (SEC) was calculated by the following equation (Eq.11):

(11) $$SEC\left(\mathrm{kWh}/kg\right)=Ec/\Delta w$$

Where Δw is change in the weight of each dried sample.

#### 2.3.5 Energy efficiency

The energy efficiency was determined by (Eq.12) [22]:

(12) $$\overset{´}{\eta }e=E_{\mathrm{em}}/SEC$$

(13) $$E_{\mathrm{em}}=lh/ec$$

Where ή_e_ is the energy efficiency (%), and *E_em_* is the energy required to evaporate moisture (kJ). Where *lh* is the latent heat of vaporization (kJ/kg) and *ec* is the energy consumed (KJ).

### 2.4 Extract Preparation

The extraction from the MO seeds powder was done by following Khan et al. [10], with slight modification. Control and pre-treated sample powder 1.5 g was homogenised in 15 ml 80 % ethanol. The mixture was subjected to vigorous agitation in a shaking incubator (Multitron II Infors SARL, Massy, France) for 30 min at 160 rpm, the homogenized was centrifuged (Centurion K1240) at 10677 × g for 10 min, after centrifugation, supernatant was carefully removed. This process were repeated 2–3 times again from the residue under the same conditions. The total extract was passed through nylon filter 0.45 μm and kept in refrigerator for further analysis.

### 2.5 Proximate

The sample's moisture and ash content were measured using a (Denver instrument IR 30 moisture content analyser) using the muffle furnace methodology, in compliance with the AOAC method [23]. The protein content was measured using a kjeldhal (Hanon, K1160), and the protein factor was 6.25. To determine the fat content, a Soxhlet technique was used. Carbohydrates (%) were calculated by subtracting the following from (100 % − moisture, fat, protein, fibre, and ash). Protein and total carbohydrate were multiplied by 4 kcal/g, whereas fat was multiplied by 9 kcal/g [10]. Each analysis was performed in replicates and reported as percentages.

### 2.6 Colour

A digital colorimeter (CR-5, Konica Japan) was used to measure colour coordinates L*, a* & b* (brightness from black to white; green to red; blue to yellow), using a standard illuminant D65 and a 10° observer and data were taken in reflectance modulus. Change in colour differences (ΔE) between germinated and control MO flour using (ΔL*^2^ + Δa*^2^ + Δb*^2^)^½^ [11], Hue angle (H) of films H^0^ = tan − 1 (b*/a*) when a* > 0 and b* > 0, and C* (Chroma) for saturation using (a*^2^ + b*^2^)^½^ & Browning index (BI) values were calculated using; BI = 100(x-0.31)/0.71, x = a*+1.75l*/5.645 l*+ a*0.301b* [10].

### 2.7 Bioactive compounds

#### 2.7.1 Total flavonoids content (TFC)

TFC were evaluated using the Jogihalli et al. [24], technique. In short, 75 uL of sodium nitrite solution (5 g/100 mL), 1.25 mL of DW & 0.25 μl of sample extract were mixed together and left to stand for 6mins. 150 μl of AlCl_3_ (10 g/100 mL) solution was added, followed by 0.5 ml of 1 mol/L NaOH and distilled water to reach 2.5 ml. Using a Spectrophotometer (GC/FT-IR, WI. 5371, Made in USA), the absorbance at 510 nm was measured. Quercetin's calibration curve was generated (y = 0.0019x + 0.0446, R^2^ = 0.997), data were expressed as mgQAE/g.

#### 2.7.2 Total phenolic content (TPC)

TPC content was calculated by using Folin-Ciocalteau methods of Lagnika et al. [25], with a slight modifications. More specifically, a vortex mixer was used to mix 200 ul of the extract solution with 1 mL Folin-Ciocalteu phenol reagent for 10 min. 800 μl of 7.5 % sodium carbonate was added, and the mixture was vortexed before being left to stand for two hours. Using a Spectrophotometer (GC/FT-IR, WI. 5371, Made in USA), the absorbance at 765 nm was measured. Gallic acid calibration curve was generated (y = 0.0018x − 0.0469, R^2^ = 0.99) data were expressed as mgGAE/g.

#### 2.7.3 Total carotenoids content (TCC)

For TCC determination the protocols of Barakat et al. [26] were used. 0.5 g MO seeds powder was extracted with 5 mL of BHT / ethanol with a ratio of 1:100 volume/ weight (100:1, v/w), in triplicates to isolate carotenoids. Afterward adding 0.5 mL of 80 % KOH, the vortex solution followed by saponification 10 min at 85◦C for. The mixture was cooled in an ice bath before being combined with 3 ml of cold deionized water. To separate the layers, 3 mL of n-Hexane was added and centrifuged at 7500 rpm for 5mins. Top yellow layer was removed and collected. Until the upper layers lost its colour, this process was repeated four times. The final capacity of each centrifuge tube was recorded after adding 12 mL of hexane. The blank was hexane and the samples were read at 450 and 503 nm. To calculate total carotenoid concentration in mg/100 g, use the equation: TCC = 4.642 × A450 − 3.091 × A503.

### 2.8 Antioxidant properties

#### 2.8.1 DPPH

DPPH was measured using Singh et al. [27], technique with slight modifications. First, 0.1 mL of MO extract was combined with 4.9 mL of DPPH (0.1 mM in ethanol). After 30 min in a dark environment at 25 °C, absorbance was measured at 517 nm in comparison to a blank that contained the same amounts of methanol and DPPH. Using the following formula, antioxidant capacity was calculated; DPPH= Control absorbance – Sample absorbance / Control absorbance × 100.

#### 2.8.2 FRAP

FRAP assay was performed according to Benzie and Strain [28] with slight modifications. The reagent (300 mmol/L acetate buffer, pH 3.6; 10 mmol/L TPTZ in 40 mmol/L HCl; 20 mmol/L FeCl3, 10:1:1 v/v) was mixed with sample extract (0.1 mL: 2.4 mL) and incubated (37 °C, 10 min). Absorbance was measured at 593 nm. A Trolox calibration curve (y = 0.4545x + 0.0259, R^2^ = 0.9984) was used to express results as μmolTE/g.

#### 2.8.3 ABTS

ABTS scavenging activity was examined with minor modifications by using the protocols of Sant'Anna et al. [29]. In short, 20 mM ABTS & 2.45 mM potassium persulfate (1:1v/v) were combined to get ABTS working solution, followed by incubation in the dark for 14–16 h. The prepared solution were checked at 753 nm absorbance by spectrophotometer to achieve (0.700 ± 0.020) by diluting the stock solution with ethanol (1:22). Absorbance was measured at 753 nm against by using 1 mL of ABTS^•^ working solution, 0.1 mL of MO extract & 3.9 mL of ethanol after 6 min were added. The findings were reported in (mg TE/g).

#### 2.8.4 Reducing power (RP)

RP as determined by followed Oyaizu's method [30]. MO extract (1 mL) was combined with potassium phosphate buffer (2.5 mL, 0.2 mol/L, pH 6.6) and potassium ferricyanide solution (2.5 mL, 1 %). After incubation (50 °C, 30 min), trichloroacetic acid (2.5 mL, 10 %) was added, and the mixture was centrifuged (1000 × g, 10 min). The supernatant (2.5 mL) was then mixed with distilled water (2.5 mL) and FeCl3 solution (0.5 mL, 0.1 %), and absorbance was measured at 700 nm. Results were expressed as mg AAE/g.

#### 2.8.5 Metal chelating activity (MCA)

MCA was measured with a slight modification, as described by Sant'Anna et al. [29]. In a mixture of 20 μL FeSO, 4 μL extract, and 5 mL distilled water, 50 (2 mmol/L) was added. The reaction started with 75 l of ferrozine (5 mmol/L). After 15 min of incubation, absorbance was measured at 562 nm against an acidified methanol blank & the results were reported as % MCA = (1- Sample absorbance values/Control values)*100.

#### 2.8.6 Antioxidant potency composite index

Overall APCI was calculated by averaging the standardized scores of five antioxidant assays (DPPH^•^, FRAP, ABTS, RP & MCA) [31]. Each assay was given equal weight, with the highest score set at 100. The APCI was calculated as the mean of the standardized scores across all five assays.

### 2.9 Peroxidase (POD) and polyphenol oxidase (PPO)

The extraction procedure for PPO and POD activities was based on Teoh et al. [32]. MO seeds powder (5 g) was homogenized with 30 mL of phosphate buffer (0.1 M, pH 6.2) and 1 g of polyvinylpyrrolidone, then centrifuged (3500 rpm, 15 min). The supernatant was used for enzyme assays. PPO activity was determined using a modified method from Sarpong et al. [33]. The reaction mixture contained supernatant (0.5 mL), phosphate buffer (2 mL, pH 6.2), and 4-methylcatechol (0.1 mL, 100 mM). Absorbance was measured at 420 nm for 5 min. POD activity was assessed based on Zhang et al. [34] with modifications. The reaction mixture consisted of extract (0.1 mL), guaiacol (0.1 mL, 4 % v/v), H2O2 (0.1 mL, 1 % v/v), and phosphate buffer (2.66 mL). Absorbance was monitored at 470 nm for 5 min.

### 2.10 Volatile compounds

GC–MS/HS-SPME were performed by following Hu et al. [35]. In short 5 g of each MO sample was placed in 20 ml septum-sealed glass vials with screw caps and mixed with 200 mL of the internal standard, 3-octanol (50 ng/ml in distilled water), and 5 g of sodium sulphate. To extract and absorb volatile chemicals, SPME fibre assembly (such as 50/30 μm DVB/CAR/PDMS, Stableflex 24 Ga, USA) was used for 30 min at 60 °C. The volatile-bearing fibres were promptly fed into the GC inlet and desorbed for five minutes at 250 °C using an automated auto-sampler.

GC analysis (7890A; Agilent Technologies, USA) was employed using a DB-Wax column (30 m x 0.32 mm x 0.5 um; Agilent, CA, USA) and an MS system (5975C; Agilent Technologies, USA). The injection port was 250 °C in splitless mode. After 6 min at 40 °C, the GC oven was heated to 100 °C at 3 °C/min, then to 230 °C at 5 °C/min, and ultimately to 230 °C for 10 min. Continuous 2.0 mL/min helium (99.999 %) was used as the carrier gas. The mass detector operated in electron impact mode at 70 eV (MS-El). Total ion chromatograms was used for peak area integration, and spectra were taken from 30 to 450 amu.

### 2.11 SEM

All samples microstructure including (Control, OG, US 10 and US 20) pre-treated before germinated dried at various temperatures was detected using a SEM (FEI, Quanta 250FEG) at the Henan University of Technology by following protocols of Khan et al., [10]. MO seed powder was placed on a SEM stub using double-sided tape putter-coated with gold (Ted Pella 108auto) & studied Cross-section of Control sample dried at 70 °C 60 °C 50 °C with 110x magnification (scale bar: 1 mm) seed powders of all tested condition at 1500X with scale bar (100 µm).

### 2.12 Statistical analysis

Analysis were done in replicate, summarized in means ± standard deviations. One-Way analysis and Tukey's test was use for analysis (p < 0.05). Significant differences were denoted by distinct letters. PCA and correlation heat maps were applied to find the effects of pre-treatments assisted germination followed by drying at different temperatures on targeted parameters and other response variables. All analyses & drawing were performed by SPSS Statistics Version 23.0 software and Origin 2021, 9.8 version, respectively.

## 3 Results and Discussion

### 3.1 Germination percentage and vigor index

**Fig. 1:**
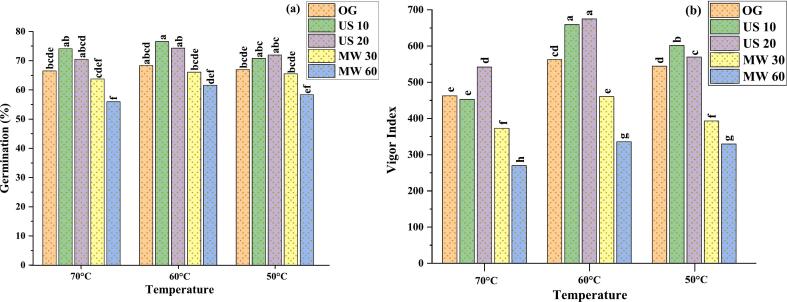
**(a-b):** Germination percentage (a) and Vigor index (b) of MO seeds. Only germinated (OG); Ultrasound 40 + 40 kHz 10 min (US 10); Ultrasound 40 + 40 kHz 20 min (US 20); Microwave 30 s (MW 30); Microwave 60 s (MW 60); Data of three replicate, (n = 3): lower case alphabets from (a-h) shows statistical differences (p ≤ 0.05).

The data presented in (Fig. 1**a)** illustrates the germination percentage of samples subjected to various treatments, including OG, US 10 & US 20 20, and MW 30 & MW 60. The OG group had a germination percentage range of 66.52 % to 68.34 %, which was consistent but not substantially higher than other treatments. US 10 showed a significant increase in seed germination, with a range of 70.81 % to 76.58 %, whereas in US 20 the germination rates was 70.52 % to 74.32 %, indicating consistent and accurate seed enhancement effects**.** Microwave exposure moderately reduced germination in the MW for 30, with values ranging from 63.76 % to 66.08 %, while MW 60 had the lowest germination rates, 55.94 % to 61.63 %, demonstrating that extended microwave treatment reduces seed germination. Furthermore, US 20 exhibited the highest vigor index 542.15 to 675.12, making this treatment the most effective, whereas the MW for 60 s group had the lowest vigor index scores 270.078 to 335.88 (Fig. 1**b**). Miano et al. [36], discovered that ultrasonic increased the germination rate without affecting vigor index or germination percentage. In addition, the seed quality may be maintained while the hydration process is improved using ultrasonic technology.

This decrease in microwave pretreatment samples is consistent with the hypothesis that thermal stress, brought on by extended exposure to microwave’s radiations, lowers seed viability. Germination inhibition might be due to embryonic damage or the temperature-induced modulation of growth metabolites such polyols, enzyme proteins, and carbs. Similarly, Tylkowska, Turek & Prieto [37], found that microwave pretreatment of common beans for 15–30 s increased germination but lowered it after 90–120 s. Pre-germinative treatments like US are essential for breaking seed dormancy, promoting quick emergence and uniform seedling development [38]. Similarly, El-Absy et al. [16], found 80.33 % and 65.33 % germination for *Moringa oleifera* seeds in the lab. Gourai et al. [39], found the maximum *Moringa oleifera* germination rates at 25 °C throughout both day and night, which served as the basis in the present research. At this specific temperature MO seeds become more active metabolically facilitating the accumulation of substrates for respiration. Our findings support Hassanein & Al-Soqeer [40], who found 90 % germination at 20 °C and no germination below 10 °C or 40 °C. This shows that temperature and pre-treatments like US and MW radiation are crucial to seed germination.

### 3.2 Drying kinetic Modelling and drying characteristics

#### 3.2.1 Moisture ratio (MR) versus drying time (DT)

**Fig. 2:**
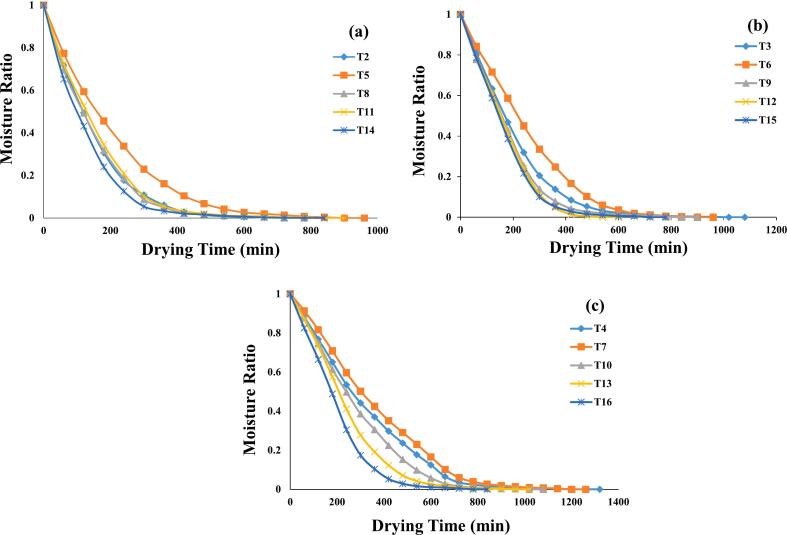
**(a-c):** Curves represent the weight loss of germinated MO seeds (n = 3): 70 °C (a); 60 °C (b) & 50 °C (c). OG at 70 °C (T2); OG at 60 °C (T3); OG at 50 °C (T4); US 10, 70 °C (T5); US 10, 60 °C (T6); US 10, 50 °C (T7); US 20, 70 °C (T8); US 20, 60 °C (T9); US 20, 50 °C (T10); MW 30, 70 °C (T11); MW 30, 60 °C (T12); MW 30, 50 °C (T13); MW 60, 70 °C (T14); MW 60, 60 °C (T15); MW 60, 50 °C (T116)..

The initial moisture content of MO seeds prior to drying ranged from 45.38 % to 52.27 %. This moisture content steadily decreased with increasing drying time across various pre-treatments and drying temperatures (50 °C to 70 °C). Each pre-treatment group exhibited different average drying times to reach a constant weight in the infrared vacuum dryer at 70 °C, 60 °C, and 50 °C. Specifically, among the pre-treated germinated groups, OG required between 660 and 1200 min, US 10 took 780 to 1140 min, US 20 required 600 to 960 min, MW 30 took 540 to 900 min and MW 60 required 660 to 920 min (Fig. 2**a-c**).

Overall, samples dried at 70 °C required 660 to 840 min, those dried at 60 °C took 480 to 960 min, while drying at 50 °C required the longest time, ranging from 720 to 1200 min, which was approximately twice that at 70 °C. Due to the increase in temperature, the moisture transfer rate to the surface is enhanced, thus accelerating the evaporation process. As a result, the drying rate increases at higher temperatures [41]. Similar results were also reported by Hao et al. [42], Jabir et al. [10], and Zhang et al. [43], for drying of grains, fruits and vegetables, respectively. In a study by Dev et al. [44], it was reported that hot air drying of Moringa oleifera seeds at similar temperatures (50, 60, and 70 °C) required approximately 1260 min to reach a final moisture content of 10 %. However, in our study, the use of infrared vacuum drying led to significantly lower final moisture content of 4.03 % to 5.28 %, with drying times ranging from 1140 to 1200 min. This comparison highlights the superior efficiency of infrared vacuum drying in achieving a lower moisture content within equal or even shorter drying times compared to conventional hot air drying.

The drying curve (Fig. 2**a)** showed a steeper slope at higher temperatures, suggesting a faster drying rate. The phenomena exhibit better water evaporation, a higher drying rate, and a shorter drying time [45]. As drying progresses into the falling rate period, internal moisture diffusion becomes more significant, as evidenced by the flattening of the drying curve. These phases are directly influenced by temperature, with higher temperatures accelerating both surface evaporation and internal moisture movement, which is captured by the increased values of the drying rate constant 'k'. These findings are consistent with a research that found that greater temperatures caused other plant species to dehydrate more quickly [46]. A higher drying temperature accelerated the reduction of MR due to increased heat supply to the seeds and enhanced moisture migration [47]. In general, the properties of the drying medium have a direct impact on the drying process. The MR vs. DT graphs revealed that MR dropped much more quickly during the first drying phase and then fell more gradually after that.

#### 3.2.2 Mathematical Modelling

Several mathematical models were assessed to get a better knowledge of the drying kinetics of the MO seeds that had germinated and were dried at different temperatures. To evaluate the quality of the fitted models, we used statistical metrics like R^2^, X^2^, RMSE, and RSS. However, multiple investigations found that R^2^ is not the sole statistical metric for selecting and evaluating nonlinear mathematical models [48,49]. Thus, X^2^, RMSE, and RSS were also considered. Considering these criteria, (Table 1**)** shows the models that presented the best adjustments for all the pre-treated geminated MO seeds dried in Infrared vacuum dryer at various temperatures.

**Table 1:** Statistical analysis of applied models on pre-treated germinated MO seeds dried under infrared vacuum dryer at various temperatures.

**Temeprature**	**70 °C**						**60 °C**							**50 °C**					
**Model**	**N/O**	**Constants & Coffi.**	**R^2^**	**X^2^**	**RMSE**	**RSS**	**N/O**	**Constants & Coffi.**	**R^2^**	**X^2^**	**RMSE**	**RSS**	**N/O**		**Constants & Coffi.**	**R^2^**	**X^2^**	**RMSE**	**RSS**
**Newton Model**	T2	k = 0.44621	0.9989	0.00202	0.00269	1.41 × 10–3	T3	k = 0.34074	0.9992	0.00037	0.00192	1.34 × 10–3	T4		k = 0.24235	0.9996	0.00011	0.00113	6.80 × 10–4
	T5	k = 0.32828	0.9999	0.00024	0.00044	5.71 × 10–5	T6	k = 0.29713	0.9999	0.00026	0.00047	6.64 × 10–5	T7		k = 0.22156	0.9999	0.00087	0.00026	3.32 × 10–5
	T8	k = 0.43822	0.9999	0.00093	0.00073	1.04 × 10–4	T9	k = 0.36789	0.9995	0.00061	0.00169	7.36 × 10–4	T10		k = 0.24828	0.9991	0.00248	0.0021	1.59 × 10–3
	T11	k = 0.42158	0.9999	0.0002	0.00015	6.10 × 10–6	T12	k = 0.44461	0.9999	0.00048	0.001	1.22 × 10–4	T13		k = 0.30619	0.9999	0.00022	0.00067	1.48 × 10–4
	T14	k = 0.47537	0.9996	0.00139	0.00145	4.76 × 10–4	T15	k = 0.41457	0.9992	0.00073	0.00231	1.04 × 10–3	T16		k = 0.35290	0.9995	0.0012	0.00179	7.24 × 10–4
**H &P Model**	T2	a = 0.49519 k = 0.33604	0.9997	0.00184	0.00138	3.74 × 10–4	T3	a = 0.96088 k = 0.35593	0.9946	0.00159	0.00499	8.99 × 10–3	T4		a = 0.89559 k = 0.26219	0.9974	0.00804	0.00323	5.53 × 10–3
	T5	a = 0.91882 k = 0.32240	0.9988	0.00112	0.0024	1.66 × 10–3	T6	a = 0.97547 k = 0.30232	0.998	0.00054	0.00346	3.47 × 10–3	T7		a = 0.97771 k = 0.22323	0.9991	0.00129	0.00206	2.06 × 10–3
	T8	a = 0.93155 k = 0.40621	0.9991	0.00306	0.0024	1.13 × 10–3	T9	a = 0.95153 k = 0.38694	0.9996	0.00133	0.00154	6.10 × 10–4	T10		a = 0.72115 k = 0.26046	0.9998	0.00258	0.00098	3.53 × 10–4
	T11	a = 0.98432 k = 0.44145	0.9979	0.00051	0.00333	2.48 × 10–3	T12	a = 0.94751 k = 0.39184	0.998	0.00467	0.00461	2.57 × 10–3	T13		a = 0.93804 k = 0.29353	0.9981	0.00223	0.00329	3.50 × 10–3
	T14	a = 0.99795 k = 0.47589	0.9989	0.00195	0.00245	1.35 × 10–3	T15	a = 1.00776 k = 0.44955	0.998	0.0015	0.00383	2.88 × 10–3	T16		a = 0.89676 k = 0.33484	0.999	0.00142	0.00266	1.59 × 10–3
**Midilli-Kucuk Model**	T2	a = 0.27839 k = 0.35713 n = 1.90908b = 0.06333	0.9999	0.00013	0.00013	3.44 × 10–6	T3	a = 0.48440 k = 0.33160 n = 1.94596b = 0.06184	0.9996	0.00551	0.00132	6.36 × 10–4	T4		a = 0.50716 k = 0.25171 n = 1.32673b = 0.04255	0.9998	0.00201	0.00065	2.24 × 10–4
	T5	a = 0.40568 k = 0.33368 n = 1.84667b = 0.06644	0.9997	0.00549	0.00123	4.39 × 10–4	T6	a = 0.54622 k = 0.33054 n = 1.65996b = 0.06098	0.9997	0.00729	0.00118	4.05 × 10–4	T7		a = 0.65289 k = 0.28352 n = 1.46681b = 0.04248	0.9998	0.00129	0.00084	3.45 × 10–4
	T8	a = 0.45067 k = 0.35106 n = 1.50822b = 0.05059	0.9999	0.00068	0.0002	1.22 × 10–5	T9	a = 0.54079 k = 0.28057 n = 1.51384b = 0.05324	0.9999	0.00236	0.00054	7.53 × 10–5	T10		a = 0.56956 k = 0.23425 n = 1.44265b = 0.04435	0.999	0.00367	0.00224	1.81 × 10–3
	T11	a = 0.52809 k = 0.32561 n = 1.61395b = 0.04694	0.9996	0.00169	0.00144	5.34 × 10–4	T12	a = 0.59284 k = 0.28812 n = 0.95713b = 0.01561	0.9998	0.00143	0.00118	1.69 × 10–4	T13		a = 0.61188 k = 0.23298 n = 1.26996b = 0.01093	0.9997	0.00184	0.00115	4.32 × 10–4
	T14	a = 0.47974 k = 0.33154 n = 1.60514b = 0.04285	0.9998	0.00107	0.00075	1.27 × 10–4	T15	a = 0.67406 k = 0.29902 n = 1.20217b = 0.01267	0.9998	0.00849	0.00114	2.54 × 10–4	T16		a = 0.65673 k = 0.20650 n = 1.24065b = 0.01185	0.9996	0.00265	0.00157	5.55 × 10–4
**Logarithmic Model**	T2	a = 0.31292 k = 0.43901c = 0.05463	0.9999	0.00128	0.00081	1.28 × 10–4	T3	a = 0.5092 k = 0.31315c = 0.06795	0.9989	0.00528	0.00224	1.81 × 10–3	T4		a = 0.94011 k = 0.21245c = 0.05107	0.9999	0.00113	0.00064	2.21 × 10–4
	T5	a = 0.62095 k = 0.28702c = 0.05527	0.9981	0.00862	0.00309	2.76 × 10–3	T6	a = 1.74985 k = 0.39227c = 0.01457	0.9996	0.00222	0.00141	5.74 × 10–4	T7		a = 0.89459 k = 0.19214c = 0.01465	0.9996	0.00189	0.00137	9.15 × 10–4
	T8	a = 0.61117 k = 0.30991c = 0.06975	0.9996	0.00246	0.00161	5.09 × 10–4	T9	a = 0.91886 k = 0.47069c = 0.01458	0.9997	0.00129	0.00109	3.06 × 10–4	T10		a = 0.96726 k = 0.24792c = 0.01760	0.9993	0.00341	0.00194	1.36 × 10–3
	T11	a = 0.48778 k = 0.40285c = 0.02491	0.9999	0.00102	0.00073	1.39 × 10–4	T12	a = 1.21246 k = 0.64459c = 0.01912	0.9998	0.00192	0.00145	2.56 × 10–4	T13		a = 0.85258 k = 0.38344c = 0.01709	0.9995	0.00296	0.00154	7.71 × 10–4
	T14	a = 0.24351 k = 0.34839c = 0.06431	0.9989	0.00433	0.00246	1.36 × 10–3	T15	a = 0.27812 k = 0.42016c = 0.01579	0.9996	0.00192	0.00171	5.76 × 10–4	T16		a = 1.03046 k = 0.28532c = 0.03380	0.9989	0.01847	0.00272	1.67 × 10–3
**Verma et al. Model**	T2	a = 0.48434 k = 0.57608 g = 0.27943	0.9954	0.00973	0.0055	5.94 × 10–3	T3	a = 0.90115 k = 0.38156 g = 0.08304	0.9969	0.00354	0.0038	5.22 × 10–3	T4		a = 0.82188 k = 0.25166 g = 0.22087	0.9988	0.00417	0.00223	2.65 × 10–3
	T5	a = 0.87016 k = 0.39597 g = 0.08928	0.9983	0.00338	0.00291	2.45 × 10–3	T6	a = 0.82403 k = 0.31091 g = 0.14723	0.9992	0.00383	0.0022	1.40 × 10–3	T7		a = 0.78735 k = 0.28105 g = 0.07075	0.9999	0.00089	0.00069	2.30 × 10–4
	T8	a = 0.85031 k = 0.50861 g = 0.28208	0.9983	0.00154	0.00331	2.16 × 10–3	T9	a = 0.84678 k = 0.39550 g = 0.22336	0.999	0.00114	0.00236	1.43 × 10–3	T10		a = 0.97308 k = 0.19384 g = 0.08873	0.9989	0.00292	0.00233	1.97 × 10–3
	T11	a = 0.90607 k = 0.37470 g = 0.24774	0.9979	0.00208	0.00338	2.93 × 10–3	T12	a = 0.83753 k = 0.38264 g = 0.26130	0.9987	0.0044	0.00372	1.67 × 10–3	T13		a = 0.63363 k = 0.29243 g = 0.26053	0.9992	0.00329	0.00211	1.44 × 10–3
	T14	a = 0.98660 k = 0.45044 g = 0.09732	0.9979	0.00663	0.00338	2.57 × 10–3	T15	a = 0.98805 k = 0.39170 g = 0.09625	0.9982	0.00502	0.00363	2.59 × 10–3	T16		a = 0.98631 k = 0.33680 g = 0.09673	0.9988	0.02019	0.00291	1.91 × 10–3
**Page Model**	T2	n = 1.06079 k = 0.38020	0.9986	0.00315	0.00301	1.78 × 10–3	T3	n = 1.03615 k = 0.31711	0.9992	0.00069	0.00191	1.31 × 10–3	T4		n = 0.94005 k = 0.28989	0.9993	0.0064	0.00167	1.47 × 10–3
	T5	n = 0.99017 k = 0.36975	0.9991	0.00765	0.00216	1.35 × 10–3	T6	n = 0.97868 k = 0.30287	0.9995	0.00097	0.00174	8.75 × 10–4	T7		n = 0.97753 k = 0.22178	0.9993	0.00062	0.00172	1.44 × 10–3
	T8	n = 0.99213 k = 0.41560	0.9989	0.00447	0.00267	1.4 × 10–3	T9	n = 1.16928 k = 0.30980	0.9993	0.00549	0.00192	9.46 × 10–4	T10		n = 1.06209 k = 0.26486	0.9984	0.00067	0.00294	3.12 × 10–3
	T11	n = 0.98356 k = 0.41459	0.9995	0.0013	0.00165	6.98 × 10–4	T12	n = 0.98187 k = 0.40303	0.999	0.00412	0.00322	1.26 × 10–3	T13		n = 0.97857 k = 0.28268	0.9987	0.00534	0.00274	2.43 × 10–3
	T14	n = 1.02594 k = 0.43027	0.999	0.00229	0.00234	1.24 × 10–3	T15	n = 0.99284 k = 0.43828	0.9987	0.00068	0.00303	1.80 × 10–3	T16		n = 0.97505 k = 0.35526	0.9988	0.0025	0.00285	1.83 × 10–3
**Two-Term Model**	T2	a = 0.08627 k1 = 0.05850 k2 = 0.01763b = 0.13814	0.998	0.00546	0.00363	2.58 × 10–3	T3	a = 0.10247 k1 = 0.06582 k2 = 0.42612b = 1.42852	0.9982	0.00265	0.00289	3.03 × 10–3	T4		a = 0.19895 k1 = 0.07296 k2 = 0.28931b = 0.99779	0.9986	0.00239	0.00241	3.09 × 10–3
	T5	a = 0.10729 k1 = 0.05974 k2 = 0.41873b = 1.29344	0.9989	0.00278	0.00239	1.65 × 10–3	T6	a = 0.23136 k1 = 0.05475 k2 = 0.38783b = 1.37073	0.9993	0.00139	0.00205	1.22 × 10–3	T7		a = 0.20513 k1 = 0.28997 k2 = 0.30627b = 1.20190	0.9998	0.00024	0.00097	4.57 × 10–4
	T8	a = 0.25085 k1 = 0.47160 k2 = 0.50063b = 0.98151	0.9991	0.00294	0.0024	1.13 × 10–3	T9	a = 0.16170 k1 = 0.06326 k2 = 0.55876b = 1.03954	0.9993	0.00185	0.00199	1.01 × 10–3	T10		a = 0.165299 k1 = 0.04624 k2 = 0.28254b = 1.09719	0.9995	0.00187	0.00163	9.68 × 10–4
	T11	a = 0.18235 k1 = 0.30381 k2 = 0.46469b = 1.00739	0.999	0.0034	0.00232	1.38-×10-3	T12	a = 0.22315 k1 = 0.01565 k2 = 0.40359b = 0.75374	0.9994	0.00338	0.00252	7.72 × 10–4	T13		a = 0.17409 k1 = 0.01115 k2 = 0.28374b = 0.83310	0.9991	0.00338	0.00227	1.68 × 10–3
	T14	a = 0.11713 k1 = 0.04446 k2 = 0.47769b = 0.97006	0.9981	0.00608	0.00323	2.35 × 10–3	T15	a = 0.15124 k1 = 0.01281 k2 = 0.39067b = 1.02511	0.9985	0.0039	0.00331	2.15 × 10–3	T16		a = 0.18664 k1 = 0.01199 k2 = 0.34315b = 0.97744	0.9989	0.00316	0.00279	1.75 × 10–3

Table 1**: Modelling applied on the average data of each temperature:** Drying Constants and Coefficients (Const. & Coffic.); Modelling applied on the mean data taken after weight loss at various temperatures 70 °C, 60 °C & 50 °C. OG at 70 °C (T2); OG at 60 °C (T3); OG at 50 °C (T4); US 10, 70 °C (T5); US 10, 60 °C (T6); US 10, 50 °C (T7); US 20, 70 °C (T8); US 20, 60 °C (T9); US 20, 50 °C (T10); MW 30, 70 °C (T11); MW 30, 60 °C (T12); MW 30, 50 °C (T13); MW 60, 70 °C (T14); MW 60, 60 °C (T15); MW 60, 50 °C (T116).

Among the all tested models; Midilli-Kucuk model the excellent fit models presenting the highest, R^2^ ≥ 0.9997, X^2^ ≤ 0.0030, RMSE ≤ 0.0010, and RSS ≤ 0.000 followed by Newton model R^2^ ≥ 0.9995, χ^2^ ≤ 0.0008, RMSE ≤ 0.0012, and RSS ≤ 0.000, Logarithmic model R^2^ ≥ 0.9994, X^2^ ≤ 0.0038, RMSE ≤ 0.0016, and RSS ≤ 0.002, Page model R^2^ ≥ 0.9990, χ^2^ ≤ 0.0031, RMSE ≤ 0.0023, and RSS ≤ 0.001, Two term model R^2^ ≥ 0.9989, X^2^ ≤ 0.0029, RMSE ≤ 0.0024, and RSS ≤ 0.003, Verma model R^2^ ≥ 0.9983, X^2^ ≤ 0.0048, RMSE ≤ 0.0029, and RSS ≤ 0.006 and Henderson and pabis model R^2^ ≥ 0.9984, X^2^ ≤ 0.0022, RMSE ≤ 0.0028, and RSS ≤ 0.005, at 70, 60 and 50 °C, respectively (Table 1**)**. Importantly, the selection of the Midilli-Kucuk model was not based solely on statistical fit, but also on its ability to physically describe the drying process of MO seeds. The model’s structure, which includes both exponential and linear terms, effectively captures the two main drying phases: an initial rapid moisture removal dominated by surface evaporation and a slower phase controlled by internal moisture diffusion.

As the drying temperature increased, the parameters ‘k’ (drying rate constant) and ‘a’ (model coefficient) also increased, indicating enhanced moisture migration due to higher molecular mobility and improved heat transfer within the seed matrix. This behaviour suggests that, at higher temperatures, moisture migrates more rapidly through internal diffusion and evaporates more quickly at the surface, leading to a steeper drying curve slope and faster drying rate. The influence of pre-treatments on phase transitions is evident from the increased drying rate constant (k) with microwave and US treatments, suggesting that these pre-treatments enhance both surface evaporation and internal moisture diffusion. Pre-treatments likely affect seed microstructure, increasing porosity and enhancing moisture mobility, which contributes to the observed faster drying rates and lower energy consumption, as demonstrated by the lower specific energy consumption observed in MW and US pre-treated seeds.

According to Ambawat [1], Midilli-Kucuk model effectively estimated the drying kinetic curves for MO. Comparable behaviour was observed for the pre-treatments assisted drying of buckwheat grains Siqueira et al. [50], mung beans [51], and sword beans [52]. The authors data confirmed that the Midilli-Kucuk model not only fits the data statistically but also provides a robust description of the drying behavior, capturing both the drying rate variations and phase transitions.

**Fig. 3:**
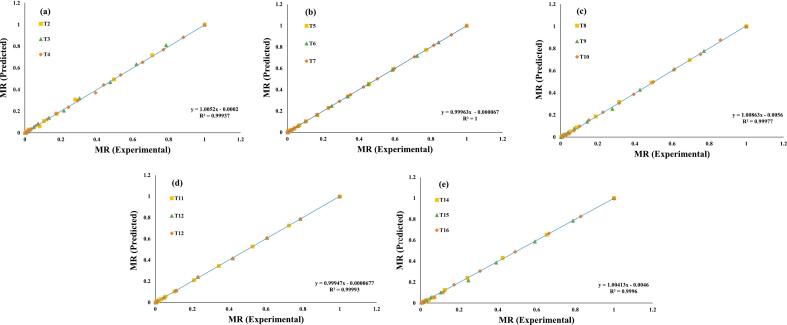
**(a-e)**: Validation of the Newton model by comparing MO seeds predicted vs. experiment data. OG (a); US 10 (b); US 20 (c); MW 30 (d); MW 60 (e); OG at 70 °C (T2); OG at 60 °C (T3); OG at 50 °C (T4); US 10, 70 °C (T5); US 10, 60 °C (T6); US 10, 50 °C (T7); US 20, 70 °C (T8); US 20, 60 °C (T9); US 20, 50 °C (T10); MW 30, 70 °C (T11); MW 30, 60 °C (T12); MW 30, 50 °C (T13); MW 60, 70 °C (T14); MW 60, 60 °C (T15); MW 60, 50 °C (T116).

**Fig. 4:**
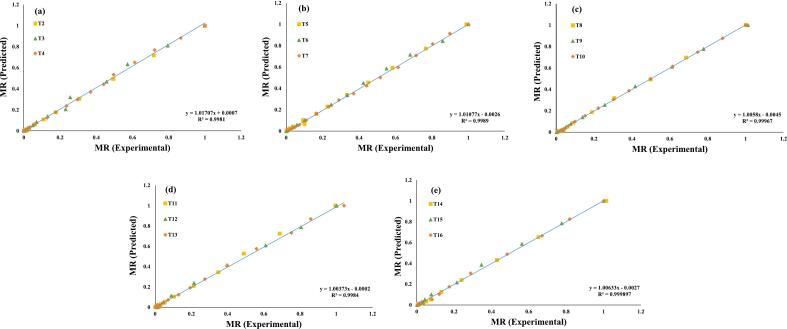
**(a-e)**: Validation of the Henderson & Pabis Model by comparing MO seeds predicted vs. experiment data. OG (a); US 10 (b); US 20 (c); MW 30 (d); MW 60 (e); OG at 70 °C (T2); OG at 60 °C (T3); OG at 50 °C (T4); US 10, 70 °C (T5); US 10, 60 °C (T6); US 10, 50 °C (T7); US 20, 70 °C (T8); US 20, 60 °C (T9); US 20, 50 °C (T10); MW 30, 70 °C (T11); MW 30, 60 °C (T12); MW 30, 50 °C (T13); MW 60, 70 °C (T14); MW 60, 60 °C (T15); MW 60, 50 °C (T116).

**Fig. 5:**
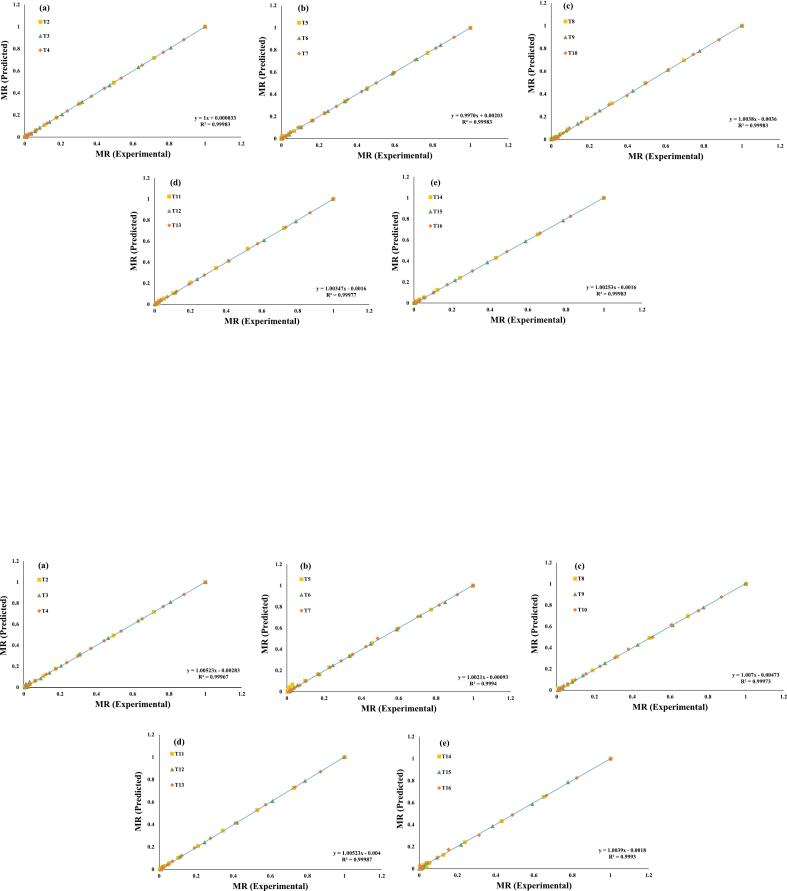
**(a-e)**: Validation of the Midilli-Kucuk Model by comparing MO seeds predicted vs. experiment data. OG (a); US 10 (b); US 20 (c); MW 30 (d); MW 60 (e); OG at 70 °C (T2); OG at 60 °C (T3); OG at 50 °C (T4); US 10, 70 °C (T5); US 10, 60 °C (T6); US 10, 50 °C (T7); US 20, 70 °C (T8); US 20, 60 °C (T9); US 20, 50 °C (T10); MW 30, 70 °C (T11); MW 30, 60 °C (T12); MW 30, 50 °C (T13); MW 60, 70 °C (T14); MW 60, 60 °C (T15); MW 60, 50 °C (T116).

**Fig. 6:**
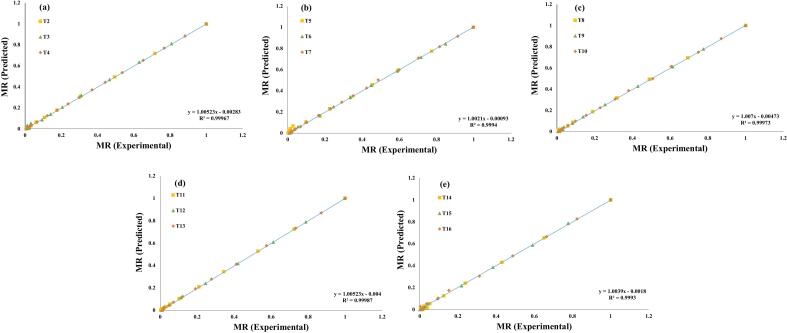
**(a-e)**: Validation of the Logarithmic Model by comparing MO seeds predicted vs. experiment data. OG (a); US 10 (b); US 20 (c); MW 30 (d); MW 60 (e); OG at 70 °C (T2); OG at 60 °C (T3); OG at 50 °C (T4); US 10, 70 °C (T5); US 10, 60 °C (T6); US 10, 50 °C (T7); US 20, 70 °C (T8); US 20, 60 °C (T9); US 20, 50 °C (T10); MW 30, 70 °C (T11); MW 30, 60 °C (T12); MW 30, 50 °C (T13); MW 60, 70 °C (T14); MW 60, 60 °C (T15); MW 60, 50 °C (T116).

**Fig. 7:**
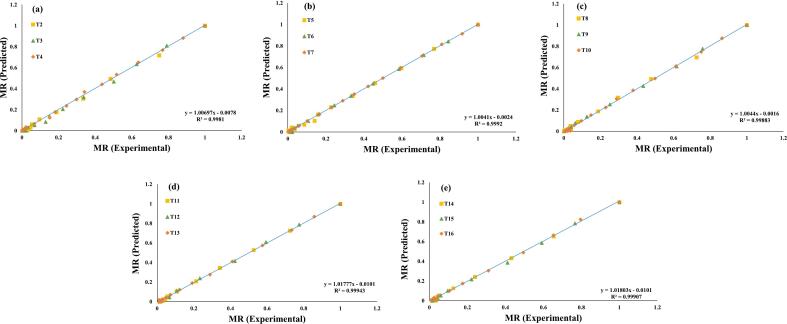
**(a-e)**: Validation of the Verma Model by comparing MO seeds predicted vs. experiment data. OG (a); US 10 (b); US 20 (c); MW 30 (d); MW 60 (e); OG at 70 °C (T2); OG at 60 °C (T3); OG at 50 °C (T4); US 10, 70 °C (T5); US 10, 60 °C (T6); US 10, 50 °C (T7); US 20, 70 °C (T8); US 20, 60 °C (T9); US 20, 50 °C (T10); MW 30, 70 °C (T11); MW 30, 60 °C (T12); MW 30, 50 °C (T13); MW 60, 70 °C (T14); MW 60, 60 °C (T15); MW 60, 50 °C (T116).

**Fig. 8:**
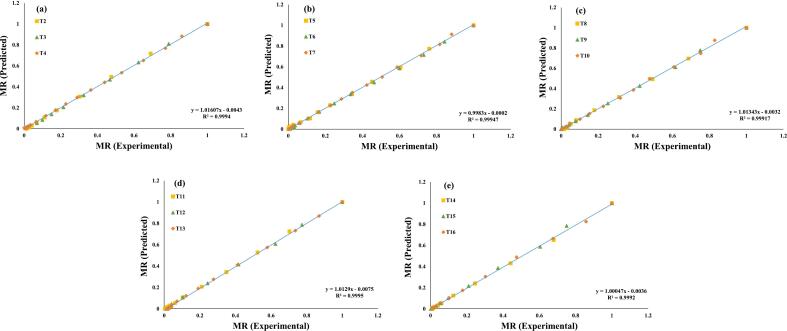
**(a-e)**: Validation of the Page Model by comparing MO seeds predicted vs. experiment data. OG (a); US 10 (b); US 20 (c); MW 30 (d); MW 60 (e); OG at 70 °C (T2); OG at 60 °C (T3); OG at 50 °C (T4); US 10, 70 °C (T5); US 10, 60 °C (T6); US 10, 50 °C (T7); US 20, 70 °C (T8); US 20, 60 °C (T9); US 20, 50 °C (T10); MW 30, 70 °C (T11); MW 30, 60 °C (T12); MW 30, 50 °C (T13); MW 60, 70 °C (T14); MW 60, 60 °C (T15); MW 60, 50 °C (T116).

**Fig. 9:**
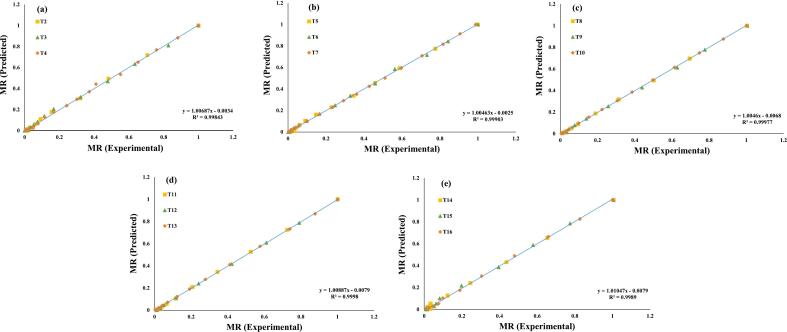
**(a-e)**: Validation of the Two-Term Model by comparing MO seeds predicted vs. experiment data. OG (a); US 10 (b); US 20 (c); MW 30 (d); MW 60 (e); OG at 70 °C (T2); OG at 60 °C (T3); OG at 50 °C (T4); US 10, 70 °C (T5); US 10, 60 °C (T6); US 10, 50 °C (T7); US 20, 70 °C (T8); US 20, 60 °C (T9); US 20, 50 °C (T10); MW 30, 70 °C (T11); MW 30, 60 °C (T12); MW 30, 50 °C (T13); MW 60, 70 °C (T14); MW 60, 60 °C (T15); MW 60, 50 °C (T116).

Additionally, these seven evaluated models were selected for a further investigation, and regression analysis was used to predict their experimental data, (Fig. 3, Fig. 4, Fig. 5, Fig. 6, Fig. 7, Fig. 8, Fig. 9) (Newton, Henderson & Pabis, Midilli-Kucuk, Logarithmic, Verma, Page, and Two-term). In the Fig. 3, Fig. 4, Fig. 5, Fig. 6, Fig. 7, Fig. 8, Fig. 9, data points were primarily grouped around the 45° straight line. This pattern provides additional confirmation of how well the models predict the drying properties of MO seeds and demonstrates their utility in characterizing moisture transfer behavior, particularly during the constant and falling rate periods. This pattern provides additional confirmation of how well the models predict the drying properties of MO seeds and demonstrates their utility in characterizing moisture transfer behaviour, particularly during the constant and falling rate periods. Thus, these broad models can be used to describe how MO seeds dry at 70, 60, and 50 °C in a vacuum air dryer. Previous investigations that used hot-air drying at US frequencies of 20, 28, 40, and 60 kHz and microwave treatments have revealed similar findings [10,53,54].

#### 3.2.3 Drying characteristics

##### 3.2.3.1 Energy consumption and water removal

According to the results, the application of pre-treatments before germination notably reduced the drying time of MO seeds during infrared vacuum drying, ranging from 6.2 to 29.5 % compared to OGS (Table 2**)**. MW pre-treatment, particularly at 30 min, was the most effective, reducing drying time by 9–29 % across drying temperatures of 50 °C, 60 °C, and 70 °C, while US pre-treatment achieved reductions of 5.4–18.4 %. This is likely due to MW's ability to disrupt seed surfaces, enhancing moisture removal. Similar results were found in studies on terebinth and turnip drying, where MW reduced drying time by 41.8–50 % and US by 7.3–21.3 % using an infrared vacuum dryer [16,55]. In terms of energy consumption, MW pre-treatment significantly reduced total energy consumption (TEC) and specific energy consumption (SEC) (p < 0.05). The lowest TEC (36.17 kWh) and SEC (8.19 kWh) were observed for MW 30 at 70 °C, while the highest TEC (71.17 kWh) and SEC (15.64 kWh) were recorded for OGS at 50 °C. Water removal efficiency was also higher for MW treatments, with MW 30 at 70 °C achieving 0.39 kWh/kg H_2_O removed, compared to 0.26 kWh/kg H_2_O removed for OGS at 50 °C. MW pre-treatment improves heat transfer through microwave energy absorption, which induces water molecule vibration, addressing thermal conductivity issues and reducing drying time [56]. This has been confirmed by Yousaf et al., who reported that MW pre-treatment resulted in the lowest energy consumption compared to US and control samples [16]. Similarly, Mierzwa et al. [57] demonstrated that MW pre-treatment led to the lowest energy consumption when drying raspberries. Moreover, Taghinezhad et al. [55] found that MW pre-treatment exhibited the lowest energy consumption in strawberries dried with infrared dryers compared to US and control treatments. Additionally, in a study by Adabi et al. [58], MW pre-treatment was shown to further reduce energy consumption across all drying methods, emphasizing its superior energy efficiency.

**Table 2:** Drying behaviour of MO seed samples under different pre-treatments and drying temperatures.

**Treatments**	**S.C**	**D.T(hrs)**	**Total Energy Consumption (kWh)**	**Specific Energy Consumption (kWh/kg)**	**Water Removed (kWh/kg H_2_O removed)/Batch**	**Energy activation (kJ/mol)**	**R^2^**	**EMD (m^2^/s)**	**R^2^**	**Drying rate (g/h)**	**ή (%)**
OG	T2	11.33	39.67 ± 2.02^fgh^	9.12 ± 1.74^ab^	0.34 ± 0.00^ab^	18.5169	0.9301	7.08 × 10^−10^	0.9831	29.22 ± 1.41^bc^	7.86 ± 0.38^bc^
	T3	18.00	63 ± 3.50^b^	11.39 ± 2.23^ab^	0.33 ± 0.00^abc^			4.83 × 10^−10^	0.9962	18.81 ± 0.72^fg^	5.06 ± 0.19^fg^
	T4	20.33	71.17 ± 2.02^a^	15.6 ± 4.22^a^	0.26 ± 0.00^c^			3.59 × 10^−10^	0.9664	12.65 ± 0.52^h^	3.4 ± 0.14^h^
US 10	T5	12.66	44.33 ± 2.02^efg^	10.02 ± 2.02^ab^	0.33 ± 0.04^abc^	12.9133	0.9853	4.74 × 10^−10^	0.9885	24.88 ± 2.20^cde^	6.69 ± 0.59^cde^
	T6	16.00	56 ± 0.00^c^	10.74 ± 0.73^ab^	0.32 ± 0.01^abc^			5.02 × 10^−10^	0.9588	20.26 ± 1.12^efg^	5.45 ± 0.30^efg^
	T7	19.33	67.67 ± 2.02^ab^	12.89 ± 1.91^ab^	0.33 ± 0.03^abc^			3.68 × 10^−10^	0.9579	17.16 ± 1.22^g^	4.62 ± 0.33^g^
US 20	T8	11.66	40.83 ± 2.02^fgh^	11.08 ± 2.26^ab^	0.34 ± 0.03^ab^	27.2067	0.9866	6.14 × 10^−10^	0.9891	28.86 ± 1.96^bc^	7.76 ± 0.52^bc^
	T9	14.66	51.33 ± 2.02^cd^	9.03 ± 2.47^ab^	0.32 ± 0.00^abc^			5.41 × 10^−10^	0.9914	21.73 ± 1.16^efg^	5.85 ± 0.31^efg^
	T10	17.66	61.83 ± 2.02^b^	10.11 ± 3.44^ab^	0.33 ± 0.04^abc^			4.84 × 10^−10^	0.9577	18.6 ± 2.45^fg^	5.01 ± 0.65^fg^
MW 30	T11	10.33	36.17 ± 2.02^h^	7.66 ± 1.09^b^	0.39 ± 0.02^a^	16.3087	0.9608	5.71 × 10^−10^	0.9912	38.18 ± 2.13^a^	10.27 ± 0.57^a^
	T12	13.00	45.5 ± 3.50d^ef^	8.44 ± 2.04^b^	0.35 ± 0.04^ab^			8.03 × 10^−10^	0.932	26.68 ± 1.08^cd^	7.18 ± 0.29^cd^
	T13	14.33	50.17 ± 4.04^cde^	9.49 ± 2.37^ab^	0.33 ± 0.00^abc^			5.18 × 10^−10^	0.983	23.12 ± 1.82^def^	6.22 ± 0.49^def^
MW 60	T14	11.00	38.5 ± 0.00^gh^	8.19 ± 2.98^b^	0.36 ± 0.02^ab^	11.7818	0.9717	6.54 × 10^−10^	0.9926	32.52 ± 1.84^b^	8.75 ± 0.49^b^
	T15	12.66	44.33 ± 2.02^efg^	9.35 ± 2.29^ab^	0.31 ± 0.02^bc^			6.35 × 10^−10^	0.9879	24.19 ± 2.40^de^	6.51 ± 0.64^de^
	T16	14.60	51.33 ± 2.02^cd^	9.81 ± 1.85^ab^	0.34 ± 0.02^ab^			5.84 × 10^−10^	0.9862	23.01 ± 2.04^def^	6.19 ± 0.55^def^

Table 2: Data are pretested in (means ± standard deviation) of three replicates (n = 3). OG at 70 °C (T2); OG at 60 °C (T3); OG at 50 °C (T4); US 10, 70 °C (T5); US 10, 60 °C (T6); US 10, 50 °C (T7); US 20, 70 °C (T8); US 20, 60 °C (T9); US 20, 50 °C (T10); MW 30, 70 °C (T11); MW 30, 60 °C (T12); MW 30, 50 °C (T13); MW 60, 70 °C (T14); MW 60, 60 °C (T15); MW 60, 50 °C (T116). Sample code (S.C); Drying temperatures (D.T); Effective moisture diffusion (EMD); Efficiency (**ή**), (a-h): shows statistical differences by Tukey’s test (p ≤ 0.05).

##### 3.2.3.2 Energy activation

**Fig. 10:**
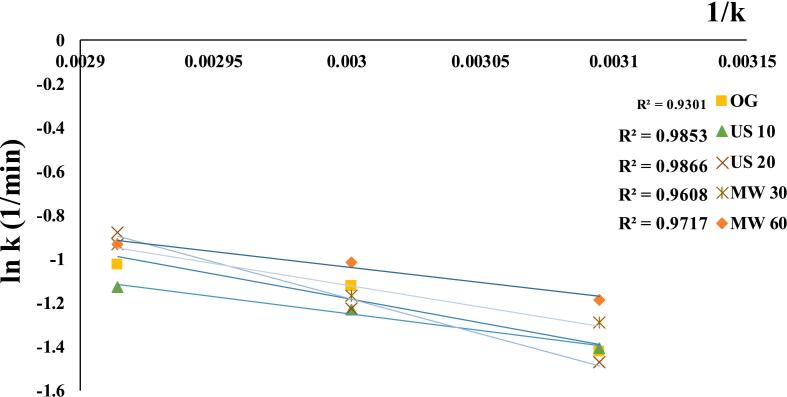
Arrhenius plot; linear connection between temperature and Ea (ln k vs slope 1/k) for Ea.

Energy activation (Ea) represents the minimum energy required to initiate moisture migration during drying. The relationship between temperature and Ea is illustrated by the Arrhenius plot shown in Fig. 10. The calculated Ea values ranged from 18.51 to 27.20 kJ/mol with R^2^ values between 0.9301 and 0.9866 (Table 2). The lowest Ea (11.78 kJ/mol) was observed in MW 60, followed by US 10 at 12.91 kJ/mol. According to Fauzi et al. [59], Food components have Ea values ranged from12.7–110 kJ/mol. Mass transfer, diffusion, and drying speeds increase with a lower Ea value because low energy is required to reach the active state. The reduction in Ea is primarily due to the structural changes induced by MW and US pre-treatments, which enhance cell wall permeability and membrane integrity by breaking down the cellular matrix and increasing permeability [60,61]. This modification enhances moisture diffusion by lowering the energy barrier for water movement. US 20 showed a higher Ea, indicating that prolonged exposure over-modified the seed structure, increasing resistance to moisture migration. In contrast, US 10 had a lower Ea, suggesting that shorter exposure optimally enhanced permeability without over-modifying the seed structure. This emphasizes the importance of US duration for efficient drying. This tendency is consistent with previous investigations [62]. Activation energy depends on pre-treatment type and duration, material, and temperature [10,19].

##### 3.2.3.3 Effective moisture diffusion (EMD)

**Fig. 11:**
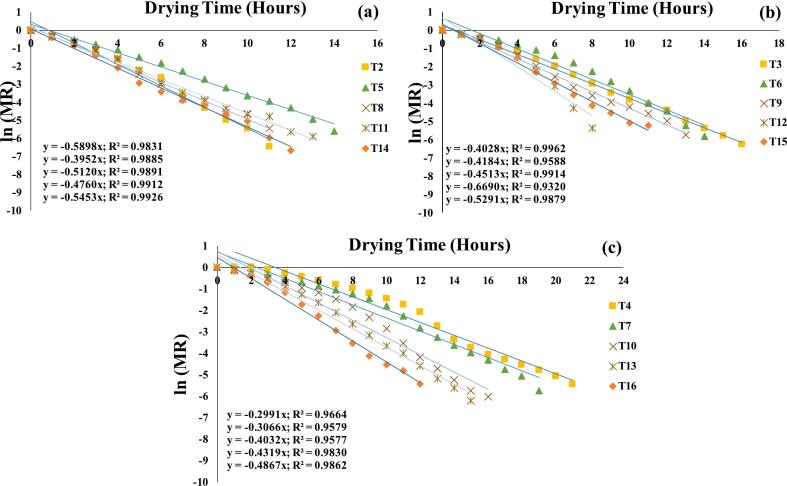
**(a-c)**: Effective moisture diffusion plots of pre-treatments assisted germinated MO seeds dried in infrared Vacuum air dryer at 70 °C (a); 60 °C (b); 50 °C (c); OG at 70 °C (T2); OG at 60 °C (T3); OG at 50 °C (T4); US 10, 70 °C (T5); US 10, 60 °C (T6); US 10, 50 °C (T7); US 20, 70 °C (T8); US 20, 60 °C (T9); US 20, 50 °C (T10); MW 30, 70 °C (T11); MW 30, 60 °C (T12); MW 30, 50 °C (T13); MW 60, 70 °C (T14); MW 60, 60 °C (T15); MW 60, 50 °C (T116).

As indicated in Table 2, the Effective Moisture Diffusivity (EMD) values ranged from 3.59 × 10^−10^ to 8.03 × 10^−10^ m^2^/s, with R^2^ 0.9320–0.9962 (Fig. 11**a-c**), which is consistent with the typical range for food materials. Madamba et al. [63] reported EMD values between 10^−9^ and 10^−11^ m^2^/s in various agricultural products, supporting our findings. The highest EMD value (8.03 × 10^−10^ m^2^/s) was observed for the microwave (MW) pre-treatment, while the lowest was recorded at 50 °C across all treatments. This variation can be attributed to structural changes induced by the pre-treatments, which enhanced moisture migration from the core to the surface, promoting faster moisture loss [10]. The increase in EMD at higher temperatures is due to enhanced molecular motion and surface suction, which facilitate water migration from the core to the surface, increasing mass transfer and reducing drying time [55]. Consistent with our findings, Moradi, Fallahi & Khaneghah [64] reported an EMD of 6.4 × 10^−10^ m^2^/s for mint leaves at 60 °C, with temperature increasing EMD due to water molecule activation, reducing viscosity, and accelerating molecular motion, thereby enhancing water migration to the surface.

Moreover, although direct experimental measurements of thermal conductivity, specific heat, porosity, and shrinkage were not conducted, these properties are essential for understanding the observed drying behavior. Literature-based interpretations suggest that US and MW pre-treatments increase porosity and open capillary structures, enhancing mass transfer rates [65]. The application of microwave energy leads to volumetric heating, improving thermal conductivity and enabling more efficient internal heat distribution [56]. This, combined with higher drying temperatures, facilitates faster heat and moisture transfer by lowering water viscosity and increasing specific heat absorption. Additionally, the relatively high drying rates observed with microwave treatments suggest minimized shrinkage and improved textural stability, further supporting efficient water removal. These factors collectively explain the improved diffusion and energy metrics seen across treatments.

##### 3.2.3.4 Drying rate and efficiency

As listed in Table 2, the highest drying rate and energy efficiency were achieved with MW pre-treatment at 70 °C, yielding a drying rate of 38.18 g/h and energy efficiency of 10.27 %. In contrast, the lowest energy efficiency was observed in the control group (OGS) at 50 °C, with a drying rate of 12.65 g/h and energy efficiency of 3.4 %. An increase in temperature enhanced energy efficiency across all treatments by improving moisture removal rates and shortening drying time, attributed to the thermal gradient between the product and drying environment, which ultimately increased drying efficiency [66]. These findings are consistent with studies on turnips [55] and blackberries [56], where higher temperatures and pre-treatments led to improved energy efficiency. Additionally, research on infrared vacuum dryers reported energy efficiency ranging from 3.76 % to 10.03 % [16], further supporting the positive relationship between temperature and energy efficiency observed in this study.

##### 3.2.3.5 Preliminary Techno-Economic Assessment

Table 3 provides a detailed Preliminary Techno-Economic Assessment associated with the processing stages. The capital cost of $5650 represents the initial investment for setting up the necessary equipment, including germination, ultrasound, microwave, and infrared drying systems. The operational cost per kg is $206.09, which accounts for expenses such as sample procurement, water consumption, and other operational activities involved in the process. The energy consumption of each stage is outlined, with pre-treatment consuming 0.825 kWh/kg, germination requiring 57.42 kWh/kg, and drying using 22.6 kWh/kg, leading to a total energy use of 80.845 kWh/kg for the complete process. Corresponding CO_2_ emissions are provided for each phase, with the total emissions for the entire process amounting to 58.6 CO_2_/kg, reflecting the environmental impact of energy consumption at each stage. The specific energy consumption (SEC) for the overall process is 43.39 kWh/kg, indicating the combined energy efficiency of all processing steps. Additional operational parameters, including germination duration (15 days), average drying time (24.17 h), and throughput (8.7 kg), offer valuable insights into the time requirements and material output. The electricity cost $0.112 per kWh, providing an estimate of ongoing operational costs. Furthermore, the system operates at 100 % capacity, indicating the full utilization of both the germination chamber and the infrared dryer during processing. These integrated metrics provide a structured and cohesive overview of the process's economic viability and environmental impact, enabling a comprehensive evaluation of its sustainability and industrial applicability.

**Table 3:** Preliminary Techno-Economic Assessment of the Processing Stages.

**Metric**	**Value**	**Unit**	**Assumption**
**Capital Cost**	5650	$	Capital investment for germination equipment, ultrasound, microwave, and infrared dryer. Initial cost to set up all the necessary machinery.
**Operational Cost per kg**	206.09	$/kg	Sample purchasing, water consumption, and other expenses.
Energy input pre-treatment	0.825	kWh/kg	Energy consumption for the pre-treatment process (US and MW).
Energy input germination	57.42	kWh/kg	Energy consumption for the germination process.
Energy input drying	22.6	kWh/kg	Energy consumption for the drying process based on dryer run by 70 °C, 60 °C, and 50 °C.
**Energy Use (Total)**	80.845	kWh/kg	Total energy consumption for the germination, drying, pre-treatment and machine run.
CO**_2_** Emissions pretreatment	0.47	CO**_2_**/kg	CO**_2_** emissions for pre-treatment (US and MW).
CO**_2_** Emissions germination	33.17	CO**_2_**/kg	CO**_2_** emissions, based on energy consumption and CO**_2_** emission factor.
CO**_2_** Emissions drying	13.1	CO**_2_**/kg	CO**_2_** emissions, calculated based on energy consumption and CO**_2_** emission factor.
**Total CO_2_ Emissions**	58.6	CO_2_/kg	Total CO_2_ emissions for the processed sample, pre-treatment, germination and drying.
**Specific Energy Consumption**	43.39	kWh/kg	Specific Energy consumption for the entire process pre-treatment, germination and drying.
**Germination Duration**	15	days	The duration for the germination process, which is 15 days.
**Average Drying Time**	24.17	hours	The average drying time based on various temperatures: 18.9 h at 70 °C, 24.77 h at 60 °C, and 28.75 h at 50 °C.
**Throughput**	8.7	kg	The sample processed: in germination and drying.
**Electricity Cost**	0.112	$	Commercial and industrial electricity cost for the germination and drying processes.
**System Load**	100	%	Germination chamber and infrared dryer, operating at full capacity (100 %).

### 3.3 Proximate composition

Table 4 illustrates the proximate of germinated MO seeds varied significantly based on the pre-treatment methods and drying temperature. The effectiveness of drying procedures depends on moisture content. Processing strategies may reduce moisture in seed flour [[67], [68]]. The lowest moisture content was 5.70 % in US 10 at 70 °C, demonstrating that US can improve drying efficiency by disrupting cell structures and boosting water evaporation. Regarding protein, germinated samples have significantly influence the content of protein in all germinated samples. US 10 resulted the highest protein increase at 60 °C (39.83), supressing all other treatments. In US 20 group the highest were found at 60 °C (39.25) and 50 °C (39.06 %) (p ≤ 0.05) (Table 4). Findings of our study, the protein content in germinated MO seeds (28.01–32.21 %) is consistent with previous studies, who reported 29.26 in control sample and a significant increase up to 33.39 % after germination of MO seeds [3]. Khan et al., [10] reported a 22.23 % increase in protein content after US assisted germination of quinoa grains. US treatment leads to the activation of specific enzymes that enhance protein synthesis by affecting cellular structures [69]. Other study reported pre-treated germinated MO seeds has protein content 57.21–70.45 % [4]. The fat content varied across treatments, the highest fat content (43.23 %) was found in OG 50 °C among all treatments. This suggests that lower drying temperatures at vacuum dryer methods may prevent the breakdown or oxidation of fats. This suggests that lower drying temperatures using vacuum dryer methods may prevent the breakdown or oxidation of fats. Our results are in line with Leone et al. [70], who found (34.7–42.4 %). Other researchers reported 28.73 to 31.9 % in germinated MO seeds [3,4,5]. The application of US treatment likely prevents the degradation of lipids, ensuring better preservation of fat content in the seeds. Furthermore, US 10 at 60 °C exhibited the highest crude fibre content (2.98 %), demonstrating that US maintains fibre by maintaining the plant matrix (Table 4). These findings show that ultrasonic treatments, especially at 60 °C US pre-treatment enhances structural integrity by breaking down the seed's cell walls through cavitation, leading to better retention of fibre content. Our findings validate the study of Saa et al. [4] who found 3.11–5.20 % crude fibre increases in pre-treated germinated MO seeds. After MO seed germination, Calizaya-Milla et al. [71], reported 2.53 to 3.08 % increase in fibre.

**Table 4:** Proximal compositions of pre-treated assisted germinated MO seeds.

**Treatments**	**Sample Code**	**Protein**	**Fat**	**Moisture**	**Crude Fibre**	**Ash**	**Carbohydrate**	**Total Energy**
**Control**	**T^1^**	35.83 ± 0.71^c^	43.23 ± 0.55^a^	4.27 ± 0.29^bc^	2.20 ± 0.071^d^	3.17 ± 0.06^c^	10.77 ± 0.89^ab^	575.49 ± 0.04^a^
**OG**	**T^2^**	38.47 ± 0.34^ab^	41.28 ± 0.61^abc^	4.35 ± 0.30^bc^	2.87 ± 0.07^ab^	3.27 ± 0.07^bc^	9.75 ± 0.37^ab^	566.27 ± 0.12^b^
	**T^3^**	39.07 ± 0.50^ab^	40.84 ± 1.51^abc^	4.03 ± 0.44^c^	2.63 ± 0.06^c^	3.54 ± 0.06^a^	9.32 ± 0.75^ab^	564.46 ± 0.02^c^
	**T^4^**	38.44 ± 0.67^ab^	41.41 ± 0.89^ab^	5.28 ± 0.43^ab^	2.65 ± 0.08^bc^	3.42 ± 0.09^ab^	9.37 ± 0.60^ab^	558.78 ± 0.13^g^
**US 10**	**T^5^**	38.01 ± 0.45^ab^	40.08 ± 0.64^bc^	5.70 ± 0.28^a^	2.21 ± 0.04^d^	3.53 ± 0.06^a^	10.48 ± 0.59^ab^	561.96 ± 0.07^d^
	**T^6^**	39.83 ± 0.51^a^	40.42 ± 0.67^bc^	4.64 ± 0.33^abc^	2.98 ± 0.12^a^	3.62 ± 0.08^a^	8.51 ± 1.07^b^	549.84 ± 0.05^o^
	**T^7^**	38.47 ± 0.46^ab^	41.14 ± 1.40^abc^	4.45 ± 0.13^bc^	2.22 ± 0.07^d^	3.61 ± 0.02^a^	10.11 ± 0.49^ab^	564.58 ± 0.09^c^
**US 20**	**T^8^**	38.05 ± 0.69^ab^	40.37 ± 0.51^bc^	4.29 ± 0.09^bc^	2.63 ± 0.04^c^	3.54 ± 0.07^a^	10.83 ± 1.01^ab^	559.99 ± 0.04^f^
	**T^9^**	39.25 ± 0.73^ab^	39.68 ± 0.42^bc^	4.73 ± 0.43^abc^	2.34 ± 0.04^d^	3.58 ± 0.06^a^	9.92 ± 0.44^ab^	555.80 ± 0.09^j^
	**T^10^**	39.06 ± 0.20^ab^	40.59 ± 1.21^bc^	4.61 ± 0.72^abc^	2.31 ± 0.08^d^	3.61 ± 0.09^a^	10.61 ± 0.67^ab^	560.85 ± 0.07^e^
**MW 30**	**T^11^**	37.77 ± 1.03^b^	39.76 ± 0.56^bc^	4.28 ± 0.36^bc^	2.76 ± 0.08^abc^	3.64 ± 0.06^a^	11.75 ± 0.40^a^	556.10 ± 0.06^i^
	**T^12^**	38.76 ± 0.77^ab^	38.76 ± 0.85^c^	4.69 ± 0.45^abc^	2.78 ± 0.06^abc^	3.26 ± 0.07^bc^	11.12 ± 0.92^a^	550.88 ± 0.05*^n^*
	**T^13^**	38.44 ± 0.58^ab^	39.60 ± 0.26^bc^	5.28 ± 0.29^ab^	2.85 ± 0.09^abc^	3.44 ± 0.07^ab^	11.06 ± 0.86^a^	551.70 ± 0.08*^m^*
**MW 60**	**T^14^**	38.17 ± 0.59^ab^	39.59 ± 0.87^bc^	4.92 ± 0.61^abc^	2.35 ± 0.04^d^	3.56 ± 0.06^a^	11.21 ± 0.76^a^	557.25 ± 0.09^h^
	**T^15^**	39.06 ± 0.75^ab^	39.58 ± 0.69^bc^	4.94 ± 0.39^abc^	2.84 ± 0.09^abc^	3.47 ± 0.07^ab^	10.13 ± 0.83^ab^	554.61 ± 0.07^k^
	**T^16^**	38.37 ± 0.64^ab^	40.45 ± 1.08^bc^	4.81 ± 0.43^abc^	2.72 ± 0.03^bc^	3.42 ± 0.08^ab^	10.41 ± 1.57^ab^	554.10 ± 0.06^l^

Table 4: Data are pretested in (means ± standard deviation) of three replicates (n = 3); proximate data expressed g/100 g, Energy kcal/100 g on dry matter basis; OG at 70 °C (T2); OG at 60 °C (T3); OG at 50 °C (T4); US 10, 70 °C (T5); US 10, 60 °C (T6); US 10, 50 °C (T7); US 20, 70 °C (T8); US 20, 60 °C (T9); US 20, 50 °C (T10); MW 30, 70 °C (T11); MW 30, 60 °C (T12); MW 30, 50 °C (T13); MW 60, 70 °C (T14); MW 60, 60 °C (T15); MW 60, 50 °C (T116). (a-i), shows statistical differences by Tukey’s test (p ≤ 0.05).

Ash content, expressing the seed's mineral content, was most significant in US 20 at 50 °C, at 3.61 %, as compared to control 3.17 %, demonstrating that moderate temperatures and long durations can increase mineral content. MW 30 at 70 °C and MW 60 at 70 °C maintained ash levels at 3.56 %, showing that short drying periods retain mineral content. US pre-treatment increases porosity, enhancing the diffusion of minerals and helping to maintain a higher mineral content. Our results are consistent with previous studies, who reported (3.17–3.62 %) ash content in MO seeds [3,71]. Notably, germination increased ash content, suggesting enhanced mineral content in MO seeds. Umerah, Asouzu & Okoye [72], reported significant increases from 2.46 % to 15.2 % after drying of germinated MO seed.

Regarding carbohydrates, the MW pre-treatment group followed by drying effectively preserved carbohydrates by reducing moisture while minimizing sugar and starch degradation, resulting in 11.75 % in MW 30 at 70 °C. US 20 at 50 °C exhibited high carbohydrate content (10.61 %) but lower than MW groups and the control sample (10.77 %). Microwaves and US are effective carbohydrate preservation technologies, especially at shorter durations or moderate temperatures, likely due to their impact on seed cell structure. Scientific studies have reported that germination decreased carbohydrate content [3], whereas pre-treatment enhanced carbohydrate content (10.56–22.67 %) [4]. Furthermore, Vhangani et al. [73] demonstrated quadratic increases in carbs and protein with microwave power, while microwave drying exhibited a significant carbohydrate improvement.

### 3.4 Color values

Table 5 demonstrates how pre-treatments and drying temperatures impact germinated MO seeds colour parameters. The brightest samples were obtained at US 10 at 70 °C (78.35), indicating lighter samples. Flour with a brightness rating closer to 100 is brighter and better for baking [74]. Our results shows that, US 10 at 60 °C exhibited the highest values of L*, OG at 70 °C had the highest redness, whereas MW 60 at 60 °C, had the lowest value a*, indicating a more balanced greenish hue. Similary Sharma, Kataria & Singh [31], found that germination treatments increased the lightness and redness and decreased the yellowness of foxtail millet due to the oxidation of pigmented compounds, which made the flour brighter. Regarding ΔE*, the Lower values indicate greater colour retention, measuring total colour change from the original. The control sample had the highest ΔE*, indicating the most colour variance. The most stable colour was seen in US 10 at 60 °C (Table 5) (p ≤ 0.05). Results reveal that, MW 30 at 60 °C followed by US 20 especially 50 °C, exhibited a lower ΔE*. Previous study suggests that dried commodities with a lower ΔE value are of higher quality [74]. The ΔE higher than 5 means the noticeable color change for the non-trained observer [75]. Thus the best case was when the ΔE was very low.

**Table 5:** Colour values of pre-treated assisted germinated MO seeds.

**Treatments**	**Sample Code**	L*	a*	b*	**Hue Angle (H**^0^**)**	ΔE*	Chroma (C*)	**Browning Index (%)**
**Control**	**T^1^**	54.09 ± 0.11*^k^*	6.83 ± 0.05^c^	10.21 ± 0.09^l^	56.22 ± 0.08*^n^*	25.66 ± 0.12^a^	12.28 ± 0.10^i^	8.01 ± 0.05^h^
**OG**	**T^2^**	51.19 ± 0.02^l^	8.29 ± 0.07^a^	14.94 ± 0.07^f^	60.97 ± 0.08^l^	18.8 ± 0.04^c^	17.09 ± 0.02^d^	13.39 ± 0.08^a^
	**T^3^**	61.96 ± 0.13^i^	6.11 ± 0.01^f^	12.93 ± 0.06^i^	64.71 ± 0.11^i^	10.65 ± 0.01^f^	14.03 ± 0.07^g^	9.34 ± 0.10d^e^
	**T^4^**	69.52 ± 0.07^d^	6.98 ± 0.10^c^	16.24 ± 0.03^cd^	66.74 ± 0.04^f^	6.61 ± 0.02^jk^	17.68 ± 0.06^c^	8.96 ± 0.01^fg^
**US 10**	**T^5^**	66.23 ± 0.02^e^	6.01 ± 0.09^f^	22.5 ± 0.04^a^	75.04 ± 0.08^a^	13.75 ± 0.02^d^	23.29 ± 0.05^a^	10.29 ± 0.10^c^
	**T^6^**	78.35 ± 0.07^a^	7.48 ± 0.10^b^	16.04 ± 0.04^d^	65 ± 0.04^h^	8.39 ± 0.05^hi^	17.7 ± 0.07^c^	8.23 ± 0.08^h^
	**T^7^**	63.2 ± 0.05^h^	6.4 ± 0.05^de^	12.57 ± 0.10^j^	63.02 ± 0.10^j^	9.34 ± 0.04^gh^	14.11 ± 0.05^g^	9.05 ± 0.04^ef^
**US 20**	**T^8^**	60.02 ± 0.12^j^	7.26 ± 0.09^b^	13.17 ± 0.09^h^	61.13 ± 0.03^l^	12.57 ± 0.05^e^	15.04 ± 0.07^f^	10.61 ± 0.09^b^
	**T^9^**	58.23 ± 0.13^jk^	5.63 ± 0.13^g^	9.89 ± 0.11*^m^*	60.35 ± 0.02*^m^*	21.08 ± 0.03^b^	11.38 ± 0.07^j^	9.49 ± 0.06^d^
	**T^10^**	71.95 ± 0.06^c^	7.48 ± 0.07^b^	16.18 ± 0.03^cd^	65.19 ± 0.16^gh^	6.01 ± 0.05^k^	17.83 ± 0.10^c^	9.59 ± 0.2^d^
**MW 30**	**T^11^**	72.18 ± 0.12^bc^	6.46 ± 0.07^d^	17.95 ± 0.10^b^	70.21 ± 0.10^b^	7.75 ± 0.06^ij^	19.08 ± 0.08^b^	10.83 ± 0.04^b^
	**T^12^**	71.91 ± 0.09^c^	6.22 ± 0.08^def^	15.81 ± 0.02^e^	68.52 ± 0.08^c^	5.64 ± 0.05^k^	16.99 ± 0.07^d^	8.24 ± 0.09^g^
	**T^13^**	63.08 ± 0.02^h^	7.48 ± 0.04^b^	16.27 ± 0.07^c^	65.31 ± 0.07^g^	10.99 ± 0.04^fg^	17.91 ± 0.07^c^	8.73 ± 0.04^h^
**MW 60**	**T^14^**	64.54 ± 0.07^f^	5.58 ± 0.08^g^	13.93 ± 0.01^g^	68.17 ± 0.04^d^	8.63 ± 0.06^hi^	15.01 ± 0.03^f^	8.97 ± 0.07^fg^
	**T^15^**	64.08 ± 0.04^g^	6.27 ± 0.15^def^	11.99 ± 0.07^k^	62.39 ± 0.03^k^	8.36 ± 0.04^hi^	13.53 ± 0.14^h^	8.19 ± 0.08^h^
	**T^16^**	64.64 ± 0.13^f^	6.16 ± 0.07^ef^	15.01 ± 0.05^f^	67.69 ± 0.06^e^	9.01 ± 0.03^h^	16.22 ± 0.05^e^	8.69 ± 0.16^g^

Table 5**:** Data are presented in (means ± standard deviation) of three replicates (n = 3). OG at 70 °C (T2); OG at 60 °C (T3); OG at 50 °C (T4); US 10, 70 °C (T5); US 10, 60 °C (T6); US 10, 50 °C (T7); US 20, 70 °C (T8); US 20, 60 °C (T9); US 20, 50 °C (T10); MW 30, 70 °C (T11); MW 30, 60 °C (T12); MW 30, 50 °C (T13); MW 60, 70 °C (T14); MW 60, 60 °C (T15); MW 60, 50 °C (T116). (a-n), shows statistical differences by Tukey’s test (p ≤ 0.05).

Additionally, the lowest BI, observed in MW 60, is indicative of reduced non-enzymatic browning, likely due to the combined effects of moderate drying temperature and controlled exposure duration. These observations reinforce the critical role of thermal regulation and pre-treatment optimization in mitigating Maillard reactions and preserving color integrity during drying. Similar conclusions have been reported by Adabi et al. [58], who found that higher drying intensities accelerate pigment degradation and browning due to elevated rates of non-enzymatic thermal reactions. Similar trends have been reported by Soontharapirakkul & Kotpat [76] for mushrooms, and Tian et al. [77] for germinated soybean.

### 3.5 Peroxidase (POD) and polyphenol oxidase (PPO)

**Fig. 12:**
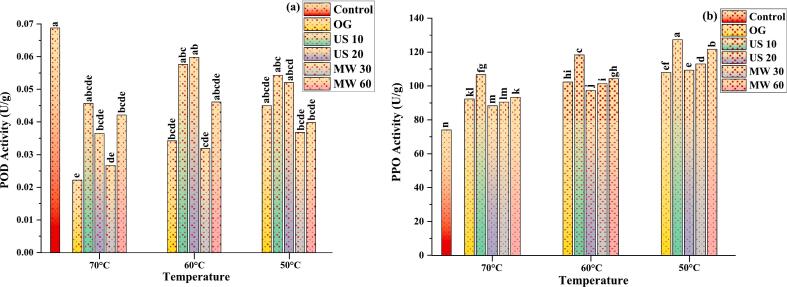
**(a-b)**: Effects of pre-treatments assisted germinated MO seeds followed by dried in infrared vacuum dryer at various temperatures. Peroxidase (POD) (a); Polyphenol oxidase (PPO) (b). Only germinated (OG); Ultrasound 40 + 40 kHz 10 min (US 10); Ultrasound 40 + 40 kHz 20 min (US 20); Microwave 30 s (MW 30); Microwave 60 s (MW 60); Data of three replicate, (a = n): shows statistical differences by Tukey’s test (p ≤ 0.05).

POD contribute to lignin formation and boost plant disease resistance by strengthening cell membranes and conveying signals to surrounding healthy cells [78]. After various pre-treatments and drying temperatures, germinated MO seeds had significantly higher POD activity than the control (Fig. 12**a**). This increase in metabolic activity supports seedling development through the release of membrane-bound enzymes [79]. Enhanced POD activity alleviates oxidative stress, promotes lignification, and protects the cell walls from lipid peroxidation and cross-linking under abiotic stress conditions. This indicates that drying temperatures influence POD activity, with 60 °C being the optimal temperature for enzyme stability, where enzyme structures remain intact, maximizing activity. Fig. 12**a** indicate that polyphenol oxidase enzyme activity increases with germination, dried at 50 °C and 60 °C, and subsequently drops at 70 °C. It is previously stated that drying at moderate temperatures retains enzyme structure and activity [80]. This explains why PPO activity is strong at these temperatures. Bhuker et al. [81], found enhanced POD activity in germinated MO seeds, approaching 3.6 times at 30 °C & 2.5 times at 35 °C. Since POD activity diminishes with temperature, it inactivates beyond 70 °C. The activity of POD in dried MO seeds exhibited significant variation, highlighting the influence of acoustic treatment followed by drying at different temperatures.

Regarding PPO, in germinated MO seeds, lower drying temperatures boost PPO activity more across all treatment groups (Fig. 12**b**). US 10 at 50 °C increased PPO the most (72.07 %), followed by MW 60 (64.41 %) and OG (45.95 %). PPO activity improved when drying temperature decreased from 70 °C to 60 °C and then to 50 °C, showing that higher temperatures damage the PPO level. The MW 30 group increased PPO activity steadily with decreasing drying temperatures, reaching 52.70 % at 50 °C. Several considerations explain why US 10 at 50 °C increased PPO activity the most. Peroxidase activity may be preserved at 50 °C (1.63 to 1.67 U/g) while at 70 °C severely degraded enzyme performance (0.62 to 0.82 U/g) (p ≤ 0.001). Other researchers have shown that high heat lowers PPO levels [82]. Rashid et al. [19] reported similar findings in their study on the effects of US pre-treatment followed by hot air drying at 60 °C, 70 °C, and 80 °C on sweet potatoes. The author found a significant reduction in POD activity (71.86 %) was observed when samples were dried at 80 °C. Furthermore, cavitation effect, which involves the creation and collapse of microbubbles in the liquid, generates significant local shear pressures that disrupt cell membranes and enhance mass transfer at this US frequency [83]. This disturbance stimulates the production of PPO enzymes, making them more accessible for activation. The optimal temperature of 50 °C combined with US treatment seems to maintain PPO activity effectively. Furthermore, Magangana et al. [84] examined the influence of pre-treatments on PPO content of fruits and found that at 60 °C, PPO concentration ranged from 5.25to 4.50, decreasing as temperature increased up to 80 °C. 2.25to 1.5; at 100 °C, 1.13 to 1.50 (P ≤ 0.05).

### 3.6 Phytochemical analysis

#### 3.6.1 Total phenolic content (TPC)

TPC are naturally produced during the growth and development of plants, and they are the main secondary metabolites in most plants [85]. They are necessary for plant germination and growth. Germinated MO seeds had a TPC of between 8.74 mgGAE/g in control and 15.73 mgGAE/g in US 20 at 60 °C (Table 6). In OG samples, TPC increased slightly 11.22 mgGAE/g to 11.42 mgGAE/g), whereas microwave treatments revealed minimal increases than OG group (p ≤ 0.001). Ultrasonication at 60 °C improved TPC level more than other pre-treatments. In agreement with Cirlini et al. [86], our TPC values ranged from 3.27-7.7 mg GAE/g after pre-treatment assisted germination. Similar results have been demonstrated in MO seeds by Tariq et al. [87] (p ≤ 0.05). In leaves, seeds, and roots, da Silva et al. [88], obtained TPC from 2.185 mg GAE/g to 3.805 mg GAE/g. Infrared vacuum drying may produce browning and oxidation events that enhance TPC in US pre-treated groups. Along with our findings, Tahmasebi et al. [89], reported ultrasonic treatment greatly enhanced TPC in roselle seeds after germination.

**Table 6:** Antioxidants properties of Pre-treated assisted geminated MO seeds.

**Treatments**	**Sample Code**	**TPC** **(mg GAE/g)**	**TFC** **(mg QAE/g)**	**TCC (mg/100 g)**	**ABTS (mM TE/g)**	**Reducing Power (mM AAE/g)**	**FRAP (mM TE/g)**	**DPPH** **(%)**	**Metal Chelating (%)**
**Control**	**T^1^**	8.74 ± 1.41^b^	2.74 ± 0.68^b^	1.24 ± 0.04^g^	23.73 ± 0.09^l^	0.23 ± 0.12^d^	3.78 ± 0.07^j^	36.52 ± 0.07^i^	26.98 ± 0.13^k^
**OG**	**T^2^**	11.39 ± 1.31^ab^	5.59 ± 1.29^b^	1.48 ± 0.07^cdef^	27.12 ± 0.04^k^	0.51 ± 0.11^d^	8.42 ± 0.08^bcd^	37.97 ± 0.03^h^	27.73 ± 0.11^j^
	**T^3^**	11.42 ± 0.82^ab^	7.2 ± 1.55^ab^	1.41 ± 0.08^defg^	33.27 ± 0.07^h^	1.41 ± 0.09^a^	8.53 ± 0.07^abc^	38.17 ± 0.04^h^	34.8 ± 0.08^b^
	**T^4^**	11.22 ± 0.41^ab^	5.84 ± 1.91^b^	1.3 ± 0.11^fg^	34.27 ± 0.08^f^	1.29 ± 0.07^a^	8.31 ± 0.07^cde^	38.1 ± 0.08^h^	30.45 ± 0.09^f^
**US 10**	**T^5^**	15.51 ± 1.01^a^	8.76 ± 1.35^ab^	1.48 ± 0.04^cdef^	32.61 ± 0.09^ij^	0.5 ± 0.11^d^	8.64 ± 0.06^ab^	38.74 ± 0.06^g^	30.36 ± 0.11^f^
	**T^6^**	15.4 ± 0.88^a^	15.8 ± 2.29^a^	1.71 ± 0.07^ab^	39.27 ± 0.05^b^	1.36 ± 0.05^a^	8.71 ± 0.09^a^	40.66 ± 0.07^a^	35.32 ± 0.07^a^
	**T^7^**	14.96 ± 0.21^a^	10.12 ± 2.55^ab^	1.61 ± 0.05^abcd^	36.82 ± 0.08^c^	1 ± 0.11^bc^	8.7 ± 0.02^a^	40.16 ± 0.08^c^	34.62 ± 0.04^b^
**US 20**	**T^8^**	15.48 ± 0.42^a^	6.09 ± 2.70^b^	1.66 ± 0.07^abc^	32.54 ± 0.06^ij^	0.46 ± 0.09^d^	7.94 ± 0.02^i^	39.54 ± 0.04^ef^	29.86 ± 0.06^g^
	**T^9^**	15.73 ± 0.62^a^	8.9 ± 0.43^ab^	1.61 ± 0.10^abcd^	41.52 ± 0.08^a^	1.46 ± 0.06^a^	7.83 ± 0.14^g^	40.39 ± 0.02^b^	32.5 ± 0.02^c^
	**T^10^**	15.28 ± 0.43^a^	7.06 ± 0.53^ab^	1.83 ± 0.09^a^	35.39 ± 0.09^d^	1.26 ± 0.02^ab^	7.29 ± 0.08^fg^	39.76 ± 0.01^d^	31.33 ± 0.04^e^
**MW 30**	**T^11^**	11.91 ± 0.13^ab^	4.94 ± 0.15^b^	1.49 ± 0.04^bcdef^	35.09 ± 0.12^e^	1.26 ± 0.09^ab^	8.4 ± 0.05^bcd^	39.51 ± 0.09^ef^	28.17 ± 0.10^i^
	**T^12^**	12.79 ± 0.06^ab^	7.7 ± 0.65^ab^	1.52 ± 0.03^bcdef^	35.3 ± 0.05^de^	1.45 ± 0.08^a^	8.69 ± 0.07^a^	40.13 ± 0.06^c^	31.64 ± 0.03^d^
	**T^13^**	12.7 ± 0.13^ab^	5.5 ± 0.90^b^	1.54 ± 0.03^bcde^	33.83 ± 0.08^g^	1 ± 0.11^bc^	8.1 ± 0.04^ef^	39.78 ± 0.09^d^	29.85 ± 0.08^g^
**MW 60**	**T^14^**	12.22 ± 0.10^ab^	4.45 ± 0.70^b^	1.33 ± 0.05^efg^	32.5 ± 0.08^j^	0.8 ± 0.05^c^	7.57 ± 0.16^h^	39.37 ± 0.08^f^	28.83 ± 0.01*^h^*
	**T^15^**	12.53 ± 0.039^ab^	5.32 ± 0.40^b^	1.47 ± 0.08^cdefg^	35.46 ± 0.10^d^	1.25 ± 0.09^ab^	8.21 ± 0.08^de^	40.24 ± 0.06^bc^	31.53 ± 0.06^de^
	**T^16^**	12.32 ± 0.07^ab^	5.08 ± 0.65^b^	1.5 ± 0.12^bcdef^	32.78 ± 0.03^i^	0.91 ± 0.11^c^	7.77 ± 0.03^gh^	39.64 ± 0.07^de^	31.44 ± 0.06^de^

Table 6: Data are pretested in (means ± standard deviation) of three replicates (n = 3). OG at 70 °C (T2); OG at 60 °C (T3); OG at 50 °C (T4); US 10, 70 °C (T5); US 10, 60 °C (T6); US 10, 50 °C (T7); US 20, 70 °C (T8); US 20, 60 °C (T9); US 20, 50 °C (T10); MW 30, 70 °C (T11); MW 30, 60 °C (T12); MW 30, 50 °C (T13); MW 60, 70 °C (T14); MW 60, 60 °C (T15); MW 60, 50 °C (T116). TPC, Total phenolic content; TFC, Total flavonoid content; TCC, Total carotenoids content: DPPH, 2,2-diphenyl-1-picrylhydrazyl radical; FRAP, Ferric reducing antioxidant power; ABTS, 2,2-azinobis(3-ethylbenzothiazoline-6-sulfonic acid). (a-k): shows statistical differences by Tukey’s test (p ≤ 0.05).

#### 3.6.2 Total flavonoid content (TFC)

The TFC of germinated MO seeds ranged from 2.74 mg QAE/g in control, to 15.8 mg QAE/g in US 10 at 60 °C (Table 6). US pre-treatment increased flavonoid extraction, having the highest amount, while OG exhibited substantial TFC increases 5.59–7.2 mgQAE/g, whereas microwave treatments showed lesser increases, especially at higher temperatures (p ≤ 0.001). Our findings agree with Buthelezi et al. [90], who showed considerable TFC increases (2.01–3.52 mgQAE/g), and Zhi et al. [85], who noticed a 34.4 % increase in TFC during *Firmiana platanifolia* seed after US assisted germination. Acoustic cavitation, mechanical stress, and additional flavonoid compounds enhance TFC in US pre-treatments [91]. US pre-treatment intermittently breaks down cellular components, making flavonoids extraction possible [92,93].

#### 3.6.3 Total carotenoid content (TCC)

The sample that was subjected to US for 20 min at 50 °C had the highest TCC value 1.83 mg/100 g than control 1.24 mg/100 g (Table 6). This represents a significant improvement over the control, which had the lowest TCC followed by US 10 at 60 °C, 1.71 mg/100 g and US 20 at 60 °C 1.61 mg/100 g (p ≤ 0.001).

Microwave treatments resulted in moderate improvements, while OG showed smaller increases. US enhances TCC by disrupting cell walls and releasing carotenoids bound to cellular structures, particularly at moderate temperatures (50 °C–60 °C). These temperatures prevent thermal degradation of carotenoids, which can occur at higher temperatures (e.g., 70 °C). Our findings align with Bhuker et al. [81], who reported that carotenoid content in MO decreases by 42.1 % in roots, 32.6 % in whole seedlings, and 26.8 % in seeds when dried at 70 °C compared to 60 °C and 50 °C. US also creates microbubbles, which help break down cell walls and enhance the extraction of chemicals with a high affinity, such as carotenoids [94]. OG and microwave treatments, while useful, lack the cavitation effect, making them less effective.

#### 3.6.4 ABTS

Table 6 shows that US 20 at 60 °C had the highest ABTS value (41.52 mg TE/g), much higher than the control, 23.73 mgTE/g, while US 10 at 60 °C, 39.27 mgTE/g, and US 20 at 50 °C, 35.39 mgTE/g, likewise showed significant increases. Microwave treatments 30 at 70 °C, 35.3 mgTE/g, followed by OG exhibiting the lowest increase. Our results are in line with Cirlini et al. [86], who found germinated MO seeds showed increased ABTS values, 6.58–41.48 mgTE/g, (P ≤ 0.05). Furthermore, US pre-treatments releases phenolic and flavonoid chemicals during cavitation, increasing antioxidant capability [95]. Moderate temperatures (50 °C–60 °C) solubilise antioxidant chemicals without degrading them. US treatment reduces polymeric phenols to smaller, more reactive molecules, enhancing ABTS activity.

#### 3.6.5 Reducing power (RP)

The sample that was subjected to US 20 at 60 °C showed the highest RP 1.46 mM AAE/g, than control 0.23 mM AAE/g. Likewise, samples subjected to US for either 10 min at 60 °C (1.36 mM AAE/g) or 20 min at 50 °C (1.26 mM AAE/g) exhibited elevated levels (Table 6). OG germination led to mino increases in reducing power, whereas microwave treatments at 60 °C, (1.45 mM AAE/g) performed effectively. Our findings are consistent with Jahan et al. [96], who reported a significant increase in RP values (from 0.3933-1.4785 mM TE/g, P ≤ 0.05). Furthermore, Manzoor et al. [97], demonstrated that the increased release of reducing agents, including phenolic compounds, during cavitation is the reason for the higher reducing power in US pre-treatments. According to Mieszczakowska-Frąc et al. [98], the bioavailability of these substances is enhanced when cells are mechanically disrupted at 50–60 °C. Microwave treatments also increase reducing power by enhancing thermal energy, which can release antioxidants, but the lack of ultrasonic disruption reduces their effectiveness.

#### 3.6.6 FRAP

Among germinated MO seeds and especially, pre-treated groups, US pre-treated sample for 10 min at 60 °C exhibited the highest FRAP value, with 8.71 mM TE/g, significantly surpassing the control (3.78 mM TE/g). US 10 at 50 °C (8.7 mM TE/g) and microwave treatment at 60 °C (8.69 mM TE/g) also demonstrated substantial increases than OG group samples (8.31 to 8.42 mM TE/g) (p < 0.001). Cirlini et al. [86], also found that germinated MO seeds had a much higher FRAP value (2.22 to 11.61 mM TE/g) (p < 0.05), which is in agreement with our results (Table 6). US pre-treatment increases the reduction of ferric ions, since it releases phenolic compounds and converts them into smaller, more bioactive forms [99]. Moderate temperatures (50 °C–60 °C) prevent thermal degradation of these antioxidants, while microwave treatments lack the mechanical disruption to achieve the same efficiency.

#### 3.6.7 DPPH

For the DPPH assay, results are expressed as the percentage quenching resulting from hydrogen donor activity. Highest activity was observed in the US pre-treated sample treated for US 10 at 60 °C, 40.66 %, compared to the control (36.52 %), followed by US 20 at 50 °C (40.16 %). Microwave treatments also showed moderate increases and OG resulted in smaller improvements (Table 6). Our results are consistent with Cirlini et al. [86] & Mubeen et al. [100], who found the DPPH% range from 48.37 to 114.85 %, while US pre-treated releases free radical-scavenging phenolic and flavonoid chemicals, which boost DPPH activity [101]. Cavitation improves antioxidant solubility and activity at moderate temperatures. Heat from microwave treatments releases antioxidants but is less efficient than US pre-treated.

#### 3.6.8 Metal chelating activity (MCA)

Table 6 demonstrated the MCA % was highest in US 10 at 60 °C, 35.32 %, significantly surpassing the control (26.98 %) and other pre-treatments. US 20 at 60 °C (32.5 %) also exhibited substantial improvements. While OG shows, (34.8 %) competitive performance, followed by microwave treatments (31.64 %). Our results are consistent with Arawande et al. [102], who observed a notable increase in metal chelating activity of MO seeds, ranging from 22.36 to 38.12. Consistent with our results, many investigations have found comparable bioactive characteristics in seeds and grains after germination [7,31,[103].

#### 3.6.9 Antioxidant potency composite index (APCI)

The various antioxidant assays exhibit variations in principles, mechanisms and experimental conditions due to which antioxidants exhibit different antioxidant potency. Overall APCI index was calculated using Seeram et al. [104], method, revealing significant variations among treatments. Notably, germinated MO seeds exhibited optimal APCIs when subjected to US 10 germination and dried at 60 °C (97.50 %). In contrast, OG samples dried at 70 °C showed the lowest APCI (73.70 %). US 10 at 60 °C germinated MO seeds displayed the highest APCI (97.5 %), closely followed by US 20 at 60 °C (96.23 %) (Table 7). APCI results were consistently higher at 60 °C than 70 °C due to drying temperature. This shows that regulated germination and drying temperatures boost antioxidant activity. Germination and pre-treatment improved APCI, as raw samples had the lowest (73.70 %). Our findings show that US 10 at 60 °C drying maximise antioxidant potency (97.50 %).

**Table 7:** Antioxidant potency composite index of studied antioxidant activities.

**Treatments**	**Sample Code**	**APCI**	**ABTS Index**	**Reducing Power Index**	**FRAP Index**	**DPPH Index**	**Metal Chelating Index**
**Control**	**T^1^**	56.55	57.16	15.98	43.42	89.81	76.37
**OG**	**T^2^**	73.7	65.33	34.7	96.63	93.37	78.5
	**T^3^**	93.39	80.13	96.57	97.85	93.86	98.53
	**T^4^**	89.2	82.55	88.35	95.21	93.69	86.21
**US 10**	**T^5^**	78.63	78.55	34.24	99.15	95.27	85.96
	**T^6^**	97.5	94.59	92.92	100	100	100
	**T^7^**	90.75	88.68	68.49	99.84	98.76	98.08
**US 20**	**T^8^**	75.11	78.38	31.73	83.66	97.22	84.55
	**T^9^**	96.23	100	100	89.82	99.32	92.02
	**T^10^**	89.79	85.25	86.07	91.16	97.77	88.7
**MW 30**	**T^11^**	88.79	84.52	86.07	96.44	97.16	79.75
	**T^12^**	94.42	85.02	99.08	99.77	98.68	89.58
	**T^13^**	85.1	81.48	68.72	92.99	97.81	84.5
**MW 60**	**T^14^**	79.62	78.27	54.56	86.84	96.81	81.62
	**T^15^**	90.65	85.41	85.38	94.22	98.95	89.26
	**T^16^**	83.43	78.94	62.55	89.17	97.47	89.05

Table 7: OG at 70 °C (T2); OG at 60 °C (T3); OG at 50 °C (T4); US 10, 70 °C (T5); US 10, 60 °C (T6); US 10, 50 °C (T7); US 20, 70 °C (T8); US 20, 60 °C (T9); US 20, 50 °C (T10); MW 30, 70 °C (T11); MW 30, 60 °C (T12); MW 30, 50 °C (T13); MW 60, 70 °C (T14); MW 60, 60 °C (T15); MW 60, 50 °C (T116).

### 3.7 Correlation heat map and principal component analysis (PCA)

**Fig. 13:**
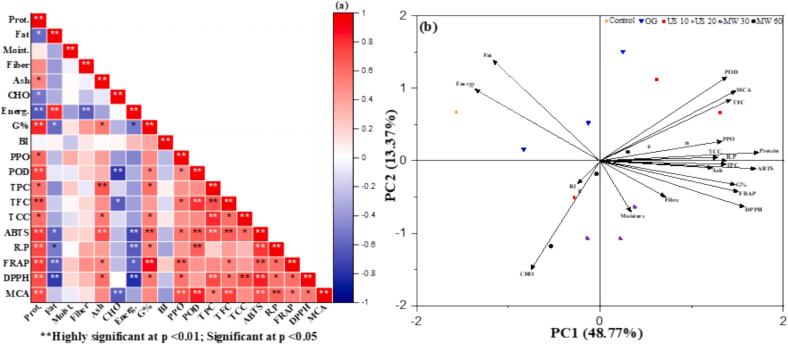
**(a-b):** Correlation Heat map (a); Loading plots of principle components 1 and 2 of the PCA proximate**,** colour values, bioactive compounds, antioxidant activities (b). Influence of pre-treated assisted germinated MO seeds followed by drying at various temperatures in infrared vacuum dryer.

The link between two variables may be expressed as a correlation coefficient (+1 & −1). If the coefficient is positive, then the variables are positively associated with one another; if it is negative, then the negatively associated with one another. A Pearson correlation matrix using the correlation coefficient (r) was produced in this investigation and shown in (Fig. 13**a**). A substantial positive connection was identified between energy and fat (0.8323) (p < 0.01**), while a significant negative correlation was detected between energy and protein (0.6657) (p < 0.01**). Ash had a substantial positive connection with protein (0.5105) (p < 0.05*). Positive correlations were found between GI and protein (0.8323) (p < 0.01**) and ash (0.5488). Significant negative correlations (0.577 & 0.516) were identified between germination % and fat and energy (p < 0.05*). Peroxidase (POD) activity correlated positively with protein (0.7614) (p < 0.01**) and negatively with carbs (0.7776) (p < 0.01**). Antioxidant actions are strongly linked to phenolic. Ash and total phenolic content (TPC) showed a considerable positive connection (0.6774) (p < 0.01**). Total flavonoid content (TFC) associated positively with protein, POD, and TPC (0.6577, 0.8173, 0.6838) (p < 0.01**), but negatively with carbs (0.6215) (p < 0.05*). ABTS antioxidant activity has a strong positive correlation with protein, ash, germination percentage, POD, TPC, and TFC (0.7805, 0.7082, 0.6327, 0.5626, 0.7032, 0.6588, 0.7046) and a significant negative correlation with energy (0.7046) (p < 0.01**). We found a substantial correlation between reducing power and protein (0.7383) and POD (0.6418) and a negative correlation with energy (0.6252) (p < 0.05*). FRAP activity associated positively with protein, germination %, PPO, and ABTS (0.7938, 0.9079, 0.6370 (p < 0.01**) and negatively with fat (0.6394) (p < 0.001**). DPPH activity associated favourably with protein (0.7114), TPC (0.7223), TCC (0.6989), and ABTS (0.8516) (p < 0.01**). Protein, PPO, POD, TFC, ABTS, and reducing power were favourably linked with metal chelation (0.7362, 0.7978, 0.8591, 0.7760, 0.767, 0.6339) (p < 0.01**). Significant positive correlations were found with TPC, FRAP, and DPPH (0.5189, 0.5264, 0.5472) (p < 0.05*), whereas carbs (0.6264) (p < 0.01**) were considerably adversely linked. Proximate and bioactive component protein content and antioxidant activity have high positive associations, suggesting a complex interaction between protein synthesis and antioxidant mechanisms in pre-treatment germinated MO seeds. As observed in Table 4 and Table 6, US pre-treatment may enhance both proximate and antioxidant production, resulting in observed correlations (Fig. 13**a**).

PCA was done to examine the relationship between pre-treated MO before germination and dried at different temperatures. Positive, negative, and no associations are shown by acute (90^◦^), straight (180^◦^), and right (90^◦^) angles between trait vectors in the (Fig. 13**b**). Proximate analysis showed that the first and second principal components, PC1 (48.77 %) and PC2 (13.37 %), contributed to the displayed data's overall variability (62.14 %). Maximum variability is explained by PC1 with an eigenvalue of 9.26 and PC2 with 0.0086. PC1 was positively correlated with Protein (0.3647), Moisture (0.0596), Fibre (0.1259), Ash (0.2162), Germination % (0.2601), PPO (0.2348), POD (0.2440), TPC (0.2418), TFC (0.22517), TCC (0.2268), ABTS (0.2995), Reducing power (0.2433), FRAP (0.2663), DPPH (0.2779), and MCA (0.2608). In PC2, it was positively co-realized with Protein (0.0364), Fat (0.4431), Energy (0.3151), PPO (0.0860), POD (0.3696), TFC (0.2705), TCC (0.0149), Reducing power (0.0013), and MCA (0.3079) and negatively co-realized with Moisture (0.2261). A PCA plot for proximities, untreated dried at MW 60 at 70 °C, followed by MW 30 at 70 °C and OG at 50 °C, caused most of the negative contribution (Fig. 13**b**). PCA loaded plot showed the association between raw and treated germinated MO seed antioxidant characteristics. DPPH, FRAP, and reducing power are closely associated and angularly closer to ABTS activity than Metal chelating (Fig. 13**a**). The biplot distance between sample coordinates is proportional to their similarity (Fig. 13**b**). Moreover, it can be observed that US pre-treated germinated MO samples exhibited higher bioactive constituents, antioxidant activities and APCI (Table 6, Table 7).

### 3.8 GC–MS analysis

The flavor and aroma of seeds play a crucial role in their sensory characteristics . GC–MS analysis of pre-treatment-assisted germinated MO seeds, using SPME fibers, identified 50 volatile compounds after excluding untraceable or impure peaks (Table 8). These compounds were categorized into nine chemical classes: 6 alcohols, 10 acids, 7 aldehydes, 6 alkanes, 2 alkenes, 6 esters, 5 ketones, 1 phenol, and 1 pyrazine. Acids were the most abundant class across all treatments, especially in MW and US treatments. Shunmugapriya et al. [105] identified 28 volatile compounds in MO, with acids as the dominant group, followed by alcohols and aldehydes, the latter notably elevated in US pre-treated samples. Consistent with previous studies, aldehydes were the dominant group in US treatments, while alkanes, ketones, and phenols were next most abundant in both control and US samples [106]. Notably, US 10 at 70 °C detected a wide range of compounds, including 1-Pentanol (17.54 %), n-Hexadecanoic acid (46 %), and *cis*-Vaccenic acid (49 %), with antioxidant potential [107]. Other effective treatments, such as MW 60 at 70 °C, detected acetic acid (5.10 %) and 1-Pentanol (15.75 %), while MW 30 at 50 °C identified long-chain fatty acids like *cis*-Vaccenic acid (49.44 %) and squalene (0.61 %) with antimicrobial and anticancer properties [108]. The optimal pre-treatment and drying temperature combinations depend on the specific volatile compounds, with lower temperatures favoring some compound detection [109].

**Table 8:** GC–MS analysis of pre-treatments assisted germination *Moringa oleifera* seeds dried at different temperatures.

**No.**	**Volatile Compound’s Name**	**Molecular Weight (amu)**	**T^1^**			**T^2^**			**T^3^**			**T^4^**		
			**PN**	**RT**	**Area%**	**PN**	**RT**	**Area%**	**PN**	**RT**	**Area%**	**PN**	**RT**	**Area%**
1	Acetic acid	60.021	**−**	**−**	**−**	5	1.685	6.9837	**−**	**−**	**−**	6	1.865	5.5437
2	3-Penten-2-one, (E)-	84.058	6	3.282	1.6631	**−**	**−**	**−**	−	−	−	−	−	−
3	1-Pentanol	88.089	8	4.854	45.8572	8	3.856	3.7333	6	3.997	3.0432	10	4.007	2.8176
4	2-Ethyl-*trans*-2-butenal	98.073	11	6.145	14.7673	10	5.959	1.1516	7	6.096	0.7704	−	−	−
5	3-Hexen-2-one	98.073	−	−	−	11	6.471	0.2514	**−**	**−**	**−**	−	−	−
6	2-Pentenal, 2-methyl-	98.073	−	−	−	−	−	−	−	−	−	12	6.115	1.0030
7	3-Penten-2-one, 4-methyl-	98.073	12	6.651	5.3498	**−**	**−**	**−**	8	6.602	0.2590	−	−	−
8	2-Hexanol, 2,3-dimethyl-	130.136	−	−	−	−	−	−	9	11.515	0.6343	−	−	−
9	3-Heptanol, 4-methyl-	130.136	16	13.842	2.7372	13	13.438	0.1871	10	13.54	0.2146	−	−	−
10	Nonanal	142.136	**−**	**−**	**−**	**−**	**−**	**−**	**−**	**−**	**−**	20	20.599	0.0923
11	Benzyl nitrile	117.058	−	−	−	20	22.464	0.1753	18	22.547	0.2580	22	22.484	0.2011
12	Cyclopentasiloxane, decamethyl-	370.094	**−**	**−**	**−**	**−**	**−**	**−**	19	23.282	0.0918	23	23.253	0.0441
13	Cyclotetrasiloxane, octamethyl-	296.075	−	−	−	**−**	**−**	**−**	23	30.006	0.0741	−	−	−
14	Benzene, (isothiocyanatomethyl)-	149.03	**−**	**−**	**−**	**−**	**−**	**−**	24	31.807	0.0768	−	−	−
15	Dodecane	170.203	**−**	**−**	**−**	−	−	−	−	−	−	24	25.254	0.0635
16	Tridecane	184.219	−	−	−	−	−	−	−	−	−	27	29.48	0.0482
17	Tetradecane	198.235	**−**	**−**	**−**	−	−	−	−	−	−	29	32.766	0.1383
18	2,4-Nonadienal, (E,E)-	138.104	30	22.581	0.3788	**−**	**−**	**−**	**−**	**−**	**−**	−	−	−
19	3-Dodecene, (Z)-	168.188	33	23.716	0.6427	**−**	**−**	**−**	**−**	**−**	**−**	−	−	−
20	2,4-Decadienal	152.12	44	29.203	6.9546	**−**	**−**	**−**	−	−	−	−	−	−
21	2-Undecenal	168.151	50	31.145	4.7110	**−**	**−**	**−**	−	−	−	−	−	−
22	5,9-Undecadien-2-one,6,10-dimethyl-,(E)-	194.167	58	34.12	0.3895	**−**	**−**	**−**	−	−	−	−	−	−
23	1-Hexanone, 1-phenyl-	176.12	59	34.358	1.9643	**−**	**−**	**−**	−	−	−	−	−	−
24	Cyclododecane	168.188	62	35.069	5.1701	**−**	**−**	**−**	−	−	−	−	−	−
25	Phenol, 2,4-bis(1,1-dimethylethyl)-	206.167	66	36.301	2.3727	**−**	**−**	**−**	−	−	−	−	−	−
26	Oxirane, hexadecyl-	268.277	75	38.399	0.6440	**−**	**−**	**−**	−	−	−	−	−	−
27	Cyclotetradecane	196.219	82	40.021	0.7148	**−**	**−**	**−**	−	−	−	−	−	−
28	*cis*-Vaccenic acid	282.256	88	41.72	0.3007	**−**	**−**	**−**	30	49.446	0.3380	−	−	−
29	Tetradecanoic acid	228.209	90	42.163	5.7721	**−**	**−**	**−**	**−**	**−**	**−**	−	−	−
30	Tetradecanal	212.214	92	42.995	0.5386	**−**	**−**	**−**	**−**	**−**	**−**	−	−	−
31	Z-8-Hexadecene	224.25	95	43.589	0.9081	**−**	**−**	**−**	**−**	**−**	**−**	**−**	**−**	**−**
32	1,2-Benzenedicarboxylic acid, bis(2 methylpropyl) ester	278.152	97	44.13	2.5424	**−**	**−**	**−**	**−**	**−**	**−**	31	44.125	0.0552
33	Pentadecanoic acid	242.225	98	44.193	2.8974	**−**	**−**	**−**	**−**	**−**	**−**	**−**	**−**	
34	Pentadecanoic acid, ethyl ester	270.256	100	44.573	1.1587	**−**	**−**	**−**	**−**	**−**	**−**	**−**	**−**	**−**
35	Dimethyl palmitamine	269.308	101	44.826	2.2580	**−**	**−**	**−**	**−**	**−**	**−**	**−**	**−**	**−**
36	Hexadecanoic acid, methyl ester	270.256	103	45.22	1.0916	**−**	**−**	**−**	**−**	**−**	**−**	**−**	**−**	**−**
37	l-(+)-Ascorbic acid 2,6-dihexadecanoate	652.491	104	45.435	1.0421	**−**	**−**	**−**	**−**	**−**	**−**	**−**	**−**	**−**
38	Palmitoleic acid	254.225	105	45.785	3.5911	25	45.775	0.1319	**−**	**−**	**−**	**−**	**−**	**−**
39	Dibutyl phthalate	278.152	106	46.009	1.8710	**−**	**−**	**−**	**−**	**−**	**−**	**−**	**−**	**−**
40	n-Hexadecanoic acid	256.24	107	46.282	12.3526	26	46.184	1.4164	28	46.165	0.3573	32	46.145	0.0968
41	Hexadecanoic acid, ethyl ester	284.272	108	46.574	24.5501	27	46.544	1.4164	29	46.544	0.0569	**−**	**−**	**−**
42	2H-Pyran-2-one, 6-heptyltetrahydro-	198.162	114	49.256	0.8026	**−**	**−**	**−**	**−**	**−**	**−**	**−**	**−**	**−**
43	9-Octadecenoic acid, (E)-	282.256	115	49.48	13.7749	28	49.563	8.6255	**−**	**−**	**−**	**−**	**−**	**−**
44	Ethyl Oleate	310.287	116	49.748	3.3924	**−**	**−**	**−**	**−**	**−**	**−**	**−**	**−**	**−**
45	Octadecanoic acid	284.272	117	49.875	7.1650	29	49.889	0.7603	**−**	**−**	**−**	**−**	**−**	**−**
46	Octadecanoic acid, ethyl ester	312.303	118	50.22	2.1870	**−**	**−**	**−**	**−**	**−**	**−**	**−**	**−**	**−**
47	Ethyl 9.cis.,11.trans.-octadecadienoate	308.272	122	51.316	1.0065	**−**	**−**	**−**	**−**	**−**	**−**	**−**	**−**	**−**
48	(R)-(−)-14-Methyl-8-hexadecyn-1-ol	252.245	124	51.847	0.5551	**−**	**−**	**−**	**−**	**−**	**−**	**−**	**−**	**−**
49	9,12-Octadecadienoic acid (Z,Z)-	280.24	126	52.363	0.4850	**−**	**−**	**−**	**−**	**−**	**−**	**−**	**−**	**−**
50	Hexanedioic acid, bis(2-ethylhexyl) ester	370.308	127	53.891	1.1125	**−**	**−**	**−**	**−**	**−**	**−**	**−**	**−**	**−**
**No.**	**Compound Name**	**Molecular Weight (amu)**		**T5**			**T6**			**T7**			**T8**	
			**PN**	**RT**	**Area%**	**PN**	**RT**	**Area%**	**PN**	**RT**	**Area%**	**PN**	**RT**	**Area%**
1	Carbon disulfide	75.944	**−**	**−**	**−**	**−**	**−**	**−**	4	1.222	2.0394	**−**	**−**	**−**
2	Acetic acid	60.021	**−**	**−**	**−**	5	1.724	6.3683	5	1.665	3.4719	**−**	**−**	**−**
3	1-Pentanol	88.089	6	4.032	17.5364	8	3.847	1.0511	8	3.817	1.6902	6	3.949	29.3023
4	2-Ethyl-*trans*-2-butenal	98.073	8	6.135	3.5737	**−**	**−**	**−**	10	5.911	0.3070	−	−	−
5	3-Hexen-2-one	98.073	−	−	−	**−**	**−**	**−**	11	6.427	0.0929	**−**	**−**	**−**
6	2-Butenal, 2-ethyl-	98.073	**−**	**−**	**−**	**−**	**−**	**−**	**−**	**−**	**−**	10	6.256	0.5893
7	2-Pentenal, 2-methyl-	98.073	−	−	−	9	5.96	0.3068	−	−	−	9	6.071	3.0866
8	3-Penten-2-one, 4-methyl-	98.073	**−**	**−**	**−**	**−**	**−**	**−**	**−**	**−**	**−**	11	6.583	0.7452
9	2-Heptanol, 2-methyl-	130.136	10	11.612	5.2616	10	11.403	0.2225	12	11.408	0.4291	**−**	**−**	**−**
10	3-Heptanol, 4-methyl-	130.136	11	13.715	0.5323	**−**	**−**	**−**	13	13.452	0.2445	14	13.632	3.5509
11	3-Nonanone	142.136	25	19.928	0.3070	**−**	**−**	**−**	**−**	**−**	**−**	**−**	**−**	**−**
12	Benzene acetonitrile, 4-hydroxy-	133.053	**−**	**−**	**−**	23	36.019	0.4655	**−**	**−**	**−**	**−**	**−**	**−**
13	Benzyl nitrile	117.058	28	22.605	0.8905	16	22.469	0.2659	21	22.479	0.0774	26	22.639	1.0026
14	Cyclopentasiloxane, decamethyl-	370.094	30	23.292	0.2458	**−**	**−**	**−**	**−**	**−**	**−**	**−**	**−**	**−**
15	5-Undecene, 6-methyl-	168.188	31	23.998	0.2618	**−**	**−**	**−**	**−**	**−**	**−**	**−**	**−**	**−**
16	2,4,7,9-Tetramethyl-5-decyn-4,7-diol	226.193	38	33.662	0.2292	**−**	**−**	**−**	**−**	**−**	**−**	**−**	**−**	**−**
17	Cyclododecane	168.188	40	35.045	0.8295	**−**	**−**	**−**	**−**	**−**	**−**	**−**	**−**	**−**
18	Phenol, 2,4-bis(1,1-dimethylethyl)-	206.167	41	36.291	0.6032	**−**	**−**	**−**	**−**	**−**	**−**	**−**	**−**	**−**
19	*cis*-Vaccenic acid	282.256	**−**	**−**	**−**	25	49.446	0.5237	27	49.436	0.1211	36	49.432	0.6582
20	n-Hexadecanoic acid	256.24	43	46.165	1.5327	24	46.15	0.2765	26	46.145	0.0704	34	46.155	1.5157
21	Hexadecanoic acid, ethyl ester	284.272	44	46.544	0.6615	**−**	**−**	**−**	**−**	**−**	**−**	35	46.544	0.6739
22	9-Octadecenoic acid, (E)-	282.256	45	49.441	1.5817				**−**	**−**	**−**	**−**	**−**	**−**
23	Ethyl Oleate	310.287	46	49.748	0.2313	**−**	**−**	**−**	**−**	**−**	**−**	**−**	**−**	**−**
24	Octadecanoic acid	284.272	47	49.85	0.2782	**−**	**−**	**−**	**−**	**−**	**−**	**−**	**−**	**−**
**No.**	**Compound Name**	**Molecular Weight (amu)**		**T9**			**T10**			**T11**			**T12**	
			**PN**	**RT**	**Area%**	**PN**	**RT**	**Area%**	**PN**	**RT**	**Area%**	**PN**	**RT**	**Area%**
1	Acetic acid	60.021	7	1.87	5.6949	5	1.875	3.3105	**−**	**−**	**−**	5	1.777	4.3602
2	1-Pentanol	88.089	11	4.027	4.3915	6	3.978	2.4253	8	3.997	12.3396	7	3.905	2.6067
3	2-Ethyl-*trans*-2-butenal	98.073	**−**	**−**	**−**	**−**	**−**	**−**	11	6.086	0.4768	8	6.047	0.4867
4	3-Hexen-2-one	98.073	**−**	**−**	**−**	**−**	**−**	**−**	**−**	**−**	**−**	10	6.558	0.1571
5	2-Pentenal, 2-methyl-	98.073	14	6.115	0.9884	8	6.115	0.6577	**−**	**−**	**−**	**−**	**−**	**−**
6	3-Penten-2-one, 4-methyl-	98.073	**−**	**−**	**−**	**−**	**−**	**−**	12	6.592	0.1954	**−**	**−**	**−**
7	2-Heptanol, 2-methyl-	130.136	17	11.495	0.4877	**−**	**−**	**−**	**−**	**−**	**−**	**−**	**−**	**−**
8	3-Heptanol, 4-methyl-	130.136	**−**	**−**	**−**	11	13.555	0.1686	14	13.525	0.1165	12	13.496	0.1481
9	n-Amyl ether	158.167	**−**	**−**	**−**	**−**	**−**	**−**	20	19.285	0.0768	**−**	**−**	**−**
10	Pyrazine, tetramethyl-	136.1	**−**	**−**	**−**	**−**	**−**	**−**	21	19.801	0.0320	**−**	**−**	**−**
11	Benzyl nitrile	117.058	25	22.557	0.1833	20	22.537	0.1068	23	22.537	0.1503	20	22.489	0.2782
12	Cyclopentasiloxane, decamethyl-	370.094	26	23.282	0.0778	21	23.268	0.0350	**−**	**−**	**−**	**−**	**−**	**−**
13	Cyclotetrasiloxane, octamethyl-	296.075	31	30.011	0.1020	**−**	**−**	**−**	**−**	**−**	**−**	**−**	**−**	**−**
14	*cis*-Vaccenic acid	282.256	37	49.441	0.3436	34	49.456	0.2363	33	49.748	0.0345	26	49.437	0.1665
15	Oleic Acid	282.256	**−**	**−**	**−**	**−**	**−**	**−**	32	49.48	0.5710	**−**	**−**	**−**
16	Hexanoic acid, 4-octyl ester	228.209	35	35.103	0.0600	**−**	**−**	**−**	**−**	**−**	**−**	**−**	**−**	**−**
17	n-Hexadecanoic acid	256.24	36	46.165	0.3349	33	46.15	0.0810	30	46.165	0.1679	25	46.15	0.1272
18	Hexadecanoic acid, ethyl ester	284.272	**−**	**−**	**−**	**−**	**−**	**−**	31	46.544	0.0508	**−**	**−**	**−**
**No.**	**Compound Name**	**Molecular Weight (amu)**		**T13**			**T14**			**T15**		**T16**		
			**PN**	**RT**	**Area%**	**PN**	**RT**	**Area%**	**PN**	**RT**	**Area%**	**PN**	**RT**	**Area%**
1	Ethanol	46.042	3	0.711	0.5190	**−**	**−**	**−**	**−**	**−**	**−**	**−**	**−**	**−**
2	Dimethyl sulfide	62.019	5	0.848	0.2649	**−**	**−**	**−**	**−**	**−**	**−**	**−**	**−**	**−**
3	Carbon disulfide	75.944	6	0.911	0.3913	**−**	**−**	**−**	4	1.432	0.8085	**−**	**−**	**−**
4	1-Propanol	60.058	7	0.969	0.9132	**−**	**−**	**−**				**−**	**−**	**−**
5	Acetic acid	60.021	8	1.349	3.4126	**−**	**−**	**−**	6	1.87	5.0954	4	1.695	3.4965
6	1-Pentanol	88.089	13	3.511	2.1071	8	4.041	15.7477	9	4.027	4.3142	5	3.851	1.5501
7	2-Ethyl-*trans*-2-butenal	98.073	15	5.599	0.3806	**−**	**−**	**−**	12	6.12	0.7455	**−**	**−**	**−**
8	3-Hexen-2-one	98.073	16	6.12	0.1012	**−**	**−**	**−**	13	6.622	0.2071	7	6.466	0.0730
9	2-Pentenal, 2-methyl-	98.073	**−**	**−**	**−**	10	6.13	2.3375	**−**	**−**	**−**	6	5.945	0.2901
10	3-Hexanol, 4-ethyl-	130.136	18	13.287	0.1802	**−**	**−**	**−**	**−**	**−**	**−**	**−**	**−**	**−**
11	2-Heptanol, 2-methyl-	130.136	17	11.232	0.4162	12	11.617	2.3230	**−**	**−**	**−**	**−**	**−**	**−**
12	3-Heptanol, 4-methyl-	130.136	**−**	**−**	**−**	13	13.71	0.8082	16	13.618	0.3147	9	13.472	0.1694
13	n-Amyl ether	158.167	**−**	**−**	**−**	**−**	**−**	**−**	**−**	**−**	**−**	15	19.217	0.0428
14	Benzyl nitrile	117.058	26	22.435	0.1185	27	22.717	0.4456	23	22.542	0.2527	17	22.493	0.1111
15	Tetradecane	198.235	**−**	**−**	**−**	**−**	**−**	**−**	27	32.771	0.0735	**−**	**−**	**−**
16	*cis*-Vaccenic acid	282.256	**−**	**−**	**−**	**−**	**−**	**−**	29	49.432	0.1187	**−**	**−**	**−**
17	Oleic Acid	282.256	**−**	**−**	**−**	40	49.431	0.2410	**−**	**−**	**−**	**−**	**−**	**−**
18	Pentanoic acid, oct-4-yl ester	214.193	**−**	**−**	**−**	**−**	**−**	**−**	26	32.401	0.0780	**−**	**−**	**−**
19	n-Hexadecanoic acid	256.24	28	46.14	0.0808	38	46.155	0.3561	28	46.15	0.2084	23	46.17	0.4441
20	Hexadecanoic acid, ethyl ester	284.272	**−**	**−**	**−**	39	46.544	0.1537	**−**	**−**	**−**	24	46.544	0.0371
21	Squalene	410.391	29	55.897	0.6107	**−**	**−**	**−**	**−**	**−**	**−**	**−**	**−**	**−**
22	9-Octadecenoic acid, (E)-	282.256	**−**	**−**	**−**	**−**	**−**	**−**	**−**	**−**	**−**	25	49.49	1.3079
23	Octadecanoic acid	284.272	**−**	**−**	**−**	**−**	**−**	**−**	**−**	**−**	**−**	26	49.865	0.1523

Table 8: 38 compounds detected in Control (T1); 11 compounds detected in OG dried at 70 °C (T2); 12 compounds detected in OG dried at 60 °C (T3); 11 compounds detected in OG dried at 50 °C (T4); 24 compounds detected in US 10 at 70 °C (T5); 8 compounds detected in US 10 at 60 °C (T6); 10 compounds detected in US 10 at 50 °C (T7); 9 compounds detected in US 20 at 70 °C (T8); 18 compounds detected in US 20 at 60 °C (T9); 8 compounds detected in MW 30 at 50 °C (T10); 11 compounds detected in MW 30 at 70 °C (T11); 8 compounds detected in MW 30 at 60 °C (T12); 23 compounds detected in MW 30 at 50 °C (T13); 8 compounds detected in MW 60 at 70 °C (T14); 11 compounds detected in MW 60 at 60 °C (T15); 11 compound detected in MW 60 at 50 °C (T16).

The observed changes in volatile compounds are driven by the combined effects of germination and pre-treatment on seed microstructure and biochemistry, promoting the release of free fatty acids (FFA) and their interaction with macromolecules [110]. This process is temperature-dependent, as higher drying temperatures accelerate reaction rates by enhancing molecular activity and enzymatic kinetics. Pre-treatments like US and MW modify seed microstructure and enzymatic activity, influencing the kinetics of volatile compound formation [111]. A key reaction contributing to the observed changes is the Millard reaction, which occurs between reducing sugars and amino acids, particularly at higher temperatures. For example, acetic acid, varying across treatments (OG at 70 °C, US 10 at 60 °C, US 20 at 60 °C, MW 30 at 60 °C, MW 60 at 60 °C), is likely a product of the Millard reaction, which is promoted in high-temperature treatments like those at 60 °C in MW and US treatments [112]. This reaction leads to the breakdown of amino acids or ester decomposition, forming acetic acid as a key volatile compound [113]. Furthermore, the presence of 1-pentanol, detected in all treatments (control, OG, US and MW), indicates lipid oxidation, with the compound suggesting lipid breakdown and alcohol formation during these treatments [114]. Additionally, 2-ethyl-*trans*-2-butenal was identified in US and MW treated samples (e.g., OG at 70 °C, US 10 at 70 °C, and MW 30 at 70 °C), with its increase at higher temperatures indicating that ester degradation enhances the formation of aldehydes like 2-ethyl-*trans*-2-butenal [115].

US pre-treated samples exhibited a greater diversity of compounds across the nine chemical classes compared to other pre-treatments, indicating that US better preserves volatile compounds at 50, 60, and 70 °C. This aligns with previous studies that show US pre-treatment significantly impacts volatile components, especially aldehydes and ketones, essential to aroma [116]. Compared to OG seeds and microwave treatments, ultrasonic treatment resulted in an immediate increase in total volatile content, consistent with findings in germinated whole brown rice [117]. Further studies on US effects on volatile profiles in roasted coffee beans found increased pyrazines and furans but decreased 2,3-pentanedione and 2-methyl pyrazine [118]. Similarly, ultrasonic treatment of Shanlan rice caused significant changes in the concentration and composition of volatile compounds, including increased ethyl hexanoate and decreased ethyl caprylate [119]. US facilitates cell wall disruption and improves the extraction of volatile compounds through cavitation, forming microbubbles that generate localized high pressure and shear forces, thereby enhancing the extraction efficiency of aromatic aldehydes and alcohols [120]. These findings indicate that volatile profiles in pre-treatment-assisted germinated MO seeds undergo distinct alterations depending on US exposure and drying conditions. This is in line with the observations of Guo et al. [121], who also found significant changes in volatile compounds during drying, emphasizing the need to explore the mechanisms behind these transformations. The current study has explored the impact of different pre-treatments on MO germinated seeds and the effects of drying on volatile compound profiles. We explicitly acknowledge that the comparisons made in this study are confined to the infrared vacuum drying method under various pre-treatments and temperatures. While hot air drying remains a well-established benchmark in drying technologies, the current study emphasizes the need for further exploration of infrared vacuum drying and its impact on volatile compounds. As supported by Rashid et al. [122], the application of US in drying processes enhances the retention of volatile compounds, although our study found that some volatiles were still lost during processing, highlighting the complexity of volatile preservation during drying. Further research is required to elucidate the detailed mechanisms underlying the formation and transformation of these volatiles during microwave- and ultrasound-assisted germination of MO seeds. Similar to our results, a study on *Flammulina velutipes* root found that vacuum- drying retained the highest content of volatile compounds, while microwave-vacuum drying produced significant levels of volatile compounds [123]. This supports our finding that different pre-treatments and drying methods, significantly affect the volatile profile, highlighting the need for further optimization of drying techniques to preserve volatile compounds effectively. Finally, Zhang et al. [124] explored the effects of various drying methods, including vacuum drying, freeze drying, and hot-air drying, on the volatile compounds of *Allium mongolicum.* Their study concluded that both vacuum drying and hot air drying effectively preserved volatile compounds, though even these well-established methods resulted in changes to the volatile profiles. This further highlights the necessity for ongoing studies to optimize drying techniques to improve the preservation of aroma and flavour, ensuring that the drying methods used are as effective as possible for maintaining the quality of the final product.

### 3.9 SEM

**Fig. 14:**
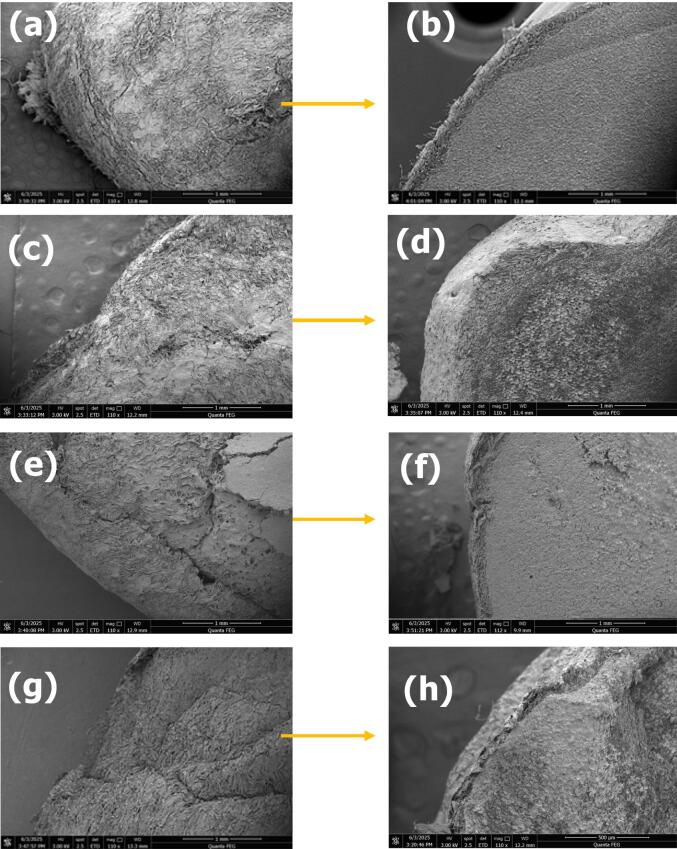
**(a-h)**: Control sample Outer layer (a); 70 °C (c); 60 °C (e); 50 °C (g); internal structure of control (b); 70 °C (d); 60 °C (f); 50 °C (h). 110X, scale bar (1 mm).

MO seeds displayed in a Cross-section, 110x with scale bar 1 mm, providing a clear view of its outer layers and inner composition affected by all drying temperatures (Fig. 14**a-h**). The control seed’s outer surface (**Fig. a**) exhibits a rough, intact seed coat, while the inner surface (Fig. 14**b**) appears smooth and compact, undamaged seed tissues. When dried at 70 °C, the outer layer (Fig. 14**c**) shows clear signs of cracking and surface roughening compared to the control, reflecting cellular shrinkage and moisture loss, while the inner layer (Fig. 14**d**) displays increased porosity and tissue disruption. These structural changes correspond with drying kinetics data that demonstrate the highest drying rates and energy efficiency at this temperature, suggesting rapid moisture removal but potential damage to seed integrity. At 60 °C most effectively preserves seed microstructure, with the outer surface (Fig. 14**e**) remaining largely intact and only minor roughening present, while the inner layer (Fig. 14**f**) remains compact and smooth. While at 50 °C, the outer surface (Fig. 14**g**) exhibits moderate shrinkage and the inner surface (Fig. 14**h**); this aligns with low drying rates, high energy consumption. Overall, the SEM observations correlate strongly with the drying kinetics, illustrating a clear temperature-dependent trade-off: as previously reported by higher drying temperatures accelerate moisture removal and enhance energy efficiency but induce significant microstructural damage, whereas moderate temperatures better maintain seed quality.

**Fig. 15:**
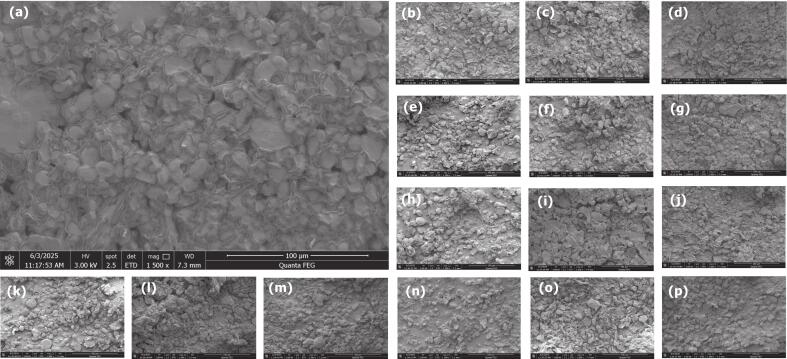
**(a-q):** Scanning electron micrographs of the MO seeds (a-p) magnificationv1500X with scale bar (100 µm). Control (a); OGS (b-d), US 10 (e-g); US 20 (h-j); MW 30 (k-m); MW 60 (n-p), germinated samples dried at 70 °C, 60 °C, and 50 °C.

The control seeds (Fig. 15**a**) exhibit a smooth, compact seed coat with prominent lipid droplets, indicative of high fat content and intact nutrient structure. Seeds subjected to only germination and dried at 70 °C, 60 °C, and 50 °C (Fig. 15 **b-d**) display minor cracking at higher temperatures, reflecting moderate structural changes consistent with drying kinetics data showing higher drying rates and energy efficiencies at elevated temperatures but potential microstructural stress. US 10 and 20 (Fig. 15 **e–**j) causes significant surface disruption and fragmentation due to cavitation effects, increasing the surface area and likely enhancing nutrient release and bioavailability, as reported in studies on quinoa, rice, and millet [10,125,126]. MW 30 and 60 (Fig. 15 **k–p**) also induce surface modifications but to a lesser extent than ultrasound. These observations correlate with drying kinetics showing that higher drying temperatures accelerate moisture removal with high drying rates and energy efficiency, indicating a temperature-dependent trade-off between drying efficiency and seed structural preservation. Lower temperatures preserve microstructure better but require longer drying times and higher energy cost as compare to sample dried at 70 °C.

## 4 Conclusion

Pre-treatment-assisted germination combined with infrared vacuum drying at 60 °C significantly improved the nutritional, structural and biochemical quality of MO seeds, with improvements closely linked to pre-treatment type and duration. US 10 resulted in the highest observed germination percentage (76.58 %) and vigor index (542.15 to 675.12) among all pre-treatments, highlighting the critical role of dual frequency and duration germination performance. Among the distinctive models explored to show the drying kinetics, the Midilli-Kucuk model proved the best fit followed by the Newton model demonstrated by highest R^2^ and lowest RMSE, RSS and X^2^ values. MW 30 at 70 °C drying significantly reduced drying time by 29 %, achieved the highest energy efficiency (10.27 %), highlighting its potential as the most energy-effective drying strategy for MO seeds. In terms of the quality attributes of MO seeds, ultrasonic pre-treatment followed by drying at 60 °C was identified as the most suitable condition, while drying at 70 °C generally resulted poor quality. Specifically, US 10 at 60 °C yielded the highest antioxidant capacity (97.50 %) accounted for 62.14 % of the total variability as revealed by PCA, and significantly enhanced POD (126.54 %) and PPO (72.07 %) activities. GC–MS analysis of pre-treatment-assisted germinated MO seeds identified 50 volatile compounds categorized into nine chemical classes, with acids being the most abundant across all treatments, particularly in the US 10 group. This underscores that optimal pre-treatment and drying temperature combinations depend on the specific volatile compounds targeted. SEM analysis confirmed that 70 °C drying caused structural degradation, whereas 60 °C preserved seed microstructure more effectively. In conclusion, the application of ultrasonic dual frequency pre-treatment assisted germination followed by infrared vacuum drying shows strong potential to enhance the nutritional quality and bioactive profile of germinated MO seeds.

## 5 Author Statement

Palwasha Gul: Conceptualization, project administration, data analysis writing and editing. Jabir Khan: Modelling, manuscript review, and editing; Yang Li, Methodology; Qingyun Li, Visualization and review; Huiyan Zhang, Methodology; Kunlun Liu: Methodology, visualization, review, and supervision. All authors have read and agreed to the published version of the manuscript.

## Funding Sources

This work was supported by the National Natural Science Foundation of China (32172259, 32472381), the Key Research and Development Project of Henan Province (231111111800) and Henan Provincial Natural Science Foundation (242300420465).

## CRediT authorship contribution statement

**Palwasha Gul:** Writing – review & editing, Writing – original draft, Project administration, Methodology, Investigation, Formal analysis, Conceptualization. **Jabir Khan:** Writing – original draft, Visualization, Validation. **Yang Li:** Methodology. **Qingyun Li:** Writing – review & editing, Visualization. **Huiyan Zhang:** Methodology. **Kunlun Liu:** Supervision, Project administration, Methodology, Conceptualization.

## Declaration of competing interest

The authors declare that they have no known competing financial interests or personal relationships that could have appeared to influence the work reported in this paper.
